# Texture Regulation of Metal–Organic Frameworks, Microwave Absorption Mechanism‐Oriented Structural Optimization and Design Perspectives

**DOI:** 10.1002/advs.202204151

**Published:** 2022-10-17

**Authors:** Zhenguo Gao, Aamir Iqbal, Tufail Hassan, Limin Zhang, Hongjing Wu, Chong Min Koo

**Affiliations:** ^1^ MOE Key Laboratory of Material Physics and Chemistry under Extraordinary Northwestern Polytechnical University Xi'an 710072 China; ^2^ School of Advanced Materials Science and Engineering SungKyunKwan University Seobu‐ro 2066, Jangan‐gu Suwon‐si Gyeonggi‐do 16419 Republic of Korea; ^3^ Materials Architecturing Research Center Korea Institute of Science and Technology (KIST) Seoul 02792 Republic of Korea; ^4^ School of Chemical Engineering SungKyunKwan University Seobu‐ro 2066, Jangan‐gu Suwon‐si Gyeonggi‐do 16419 Republic of Korea

**Keywords:** electromagnetic wave absorption, metal–organic frameworks (MOFs), optimization engineering, texture regulation

## Abstract

Texture regulation of metal–organic frameworks (MOFs) is essential for controlling their electromagnetic wave (EMW) absorption properties. This review systematically summarizes the recent advancements in texture regulation strategies for MOFs, including etching and exchange of central ions, etching and exchange of ligands, chemically induced self‐assembly, and MOF‐on‐MOF heterostructure design. Additionally, the EMW absorption mechanisms in approaches based on structure–function dependencies, including nano‐micro topological engineering, defect engineering, interface engineering, and hybrid engineering, are comprehensively explored. Finally, current challenges and future research orientation are proposed. This review aims to provide new perspectives for designing MOF‐derived EMW‐absorption materials to achieve essential breakthroughs in mechanistic investigations in this promising field.

## Graphical abstract

A novel review summarizes texture regulation of metal–organic frameworks (MOFs), including etching and exchange of central ions, etching and exchange of ligands, chemically‐induced self‐assembly, and MOF‐on‐MOF heterostructures. Relative optimization engineering toward microwave absorption mechanism is proposed, including nano‐micro topology engineering, defect engineering, interface engineering, and hybrid engineering. A perspective for future development of MOFs derived microwave absorption materials is proposed.

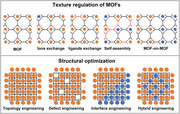

## 1 Introduction

The accelerating developments in network entity systems and the Internet of Things (IoT) have revolutionized electronic communication, intelligent detection, and modern medical science.^[^ ^1^ ^]^ However, undesirable electromagnetic (EM) radiations are generated in substantial amount during operation of these devices, which inevitably interfere with the normal response of electronic components, the accuracy of information detection, and even human health.^[^ ^2^, ^3^ ^]^ Harmful EM wave (EMW) radiation should be eliminated or attenuated. Therefore, developing microwave absorbing materials (MAMs) is becoming a hot area of research to solve this problem.

**Figure 1:**
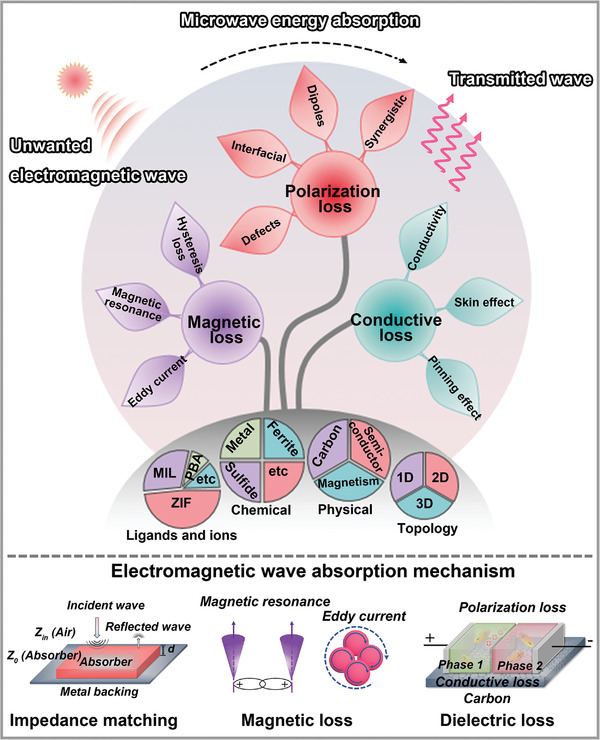
Schematics of MOFs and their derivatives with diverse structures, and illustration of conventional EMW absorption mechanisms.

Metal–organic frameworks (MOFs) derived MAMs are attracting considerable attention because of their nanostructure tunability that can control EMW absorption properties, such as structure‐induced dielectric and magnetic loss.^[^ ^4^ ^]^ MOFs are hybrids composed of metal ions and organic ligands with periodic coordination.^[^ ^5^ ^]^ As shown in **Figure** **1**, a wide variety of MOFs has been prepared and applied in EMW absorption owing to their diverse components and structures.^[^ ^6^, ^7^ ^]^ In particular, MOFs can be employed as ideal precursors for carbon‐based MAMs after pyrolysis,^[^ ^8^, ^9^, ^10^ ^]^ and can thereby control chemical, crystal, and morphological structures for tuning their metallic, semiconductor, magnetic, and even synergistic properties.^[^ ^11^ ^]^ Generally, the metal ions and ligands determine the chemical composition of the final product. For example, the common zeolitic imidazolate framework (ZIF) materials typically take Zn or Co as the central ions,^[^ ^12^, ^13^, ^14^, ^15^, ^16^, ^17^ ^]^ while the Universitetet i Oslo (UiO),^[^ ^18^, ^19^ ^]^ Prussian blue (PB)^[^ ^20^, ^21^ ^]^ and Material Institute Lavoisier (MIL)^[^ ^22^, ^23^ ^]^ MOFs generally contain Zr,^[^ ^24^ ^]^ Fe,^[^ ^25^ ^]^ and Cr,^[^ ^26^ ^]^ respectively. The topology of MOFs (1D,^[^ ^27^, ^28^ ^]^ 2D,^[^ ^29^, ^30^, ^31^ ^]^ and 3D,^[^ ^32^, ^33^, ^34^ ^]^) can not only be affected by the type of MOF, but also chemical or physical factors, such as solvent,^[^ ^35^ ^]^ surfactant,^[^ ^36^ ^]^ and temperature. Further processing of MOF precursors can achieve control over the chemical and physical features of products, such as semiconductor and magnetic properties of metal alloys,^[^ ^37^, ^38^ ^]^ ferrites,^[^ ^39^, ^40^ ^]^ metal sulfides,^[^ ^41^ ^]^ and others.^[^ ^42^ ^]^ Therefore, MOF‐derived MAMs are considered as efficient materials owing to their aforementioned unique structure and performance.

Basically, texture refers to the statistically preferred orientation of individual grains in a polycrystal. Herein, most MOFs are typical polycrystals with subtle long‐range ordering. In this review, the “texture” is defined as not only the crystal order characteristics but also the final microstructure of MOFs. The texture of MOFs mainly depends on their basic components (metal ions and ligands), and chemical and physical conditions during deposition. The texture regulation of MOFs has become the most promising and direct mean of manipulating the chemical components and configurations of MAMs, apart from composite fabrication. Texture regulation further expands the variety of MOFs, thereby providing ample options for obtaining MAMs with specific EM responsiveness. Although, efficient strategies have been devised for developing highly efficient MOF‐derived MAMs, mechanistic analysis of EMW absorption in terms of engineering optimization has not been comprehensively discussed yet. Therefore, in‐depth understanding of the texture‐performance relationships in MOF‐derived MAMs and their intrinsic EMW absorption properties from a mechanistic perspective is lacking.

Therefore, the texture regulation of MOFs for realizing high‐performance EMW absorption is systematically and comprehensively summarized in this review from both methodological and mechanistic perspectives. First, strategies for preparing MOF‐based MAMs, including etching and exchange of central ions, etching and exchange of ligands, chemically induced self‐assembly, and MOF‐on‐MOF heterostructure design, are summarized. Second, EMW absorption mechanisms of MAMs with different texture designs achieved through nano‐micro topology engineering, defect engineering, interface engineering, and hybrid engineering are comprehensively discussed. Finally, current challenges, feasible solutions, and prospective research directions for rational texture regulation of MOFs are proposed with the aim of realizing advanced MAMs.

## 2 Strategies for Synthesizing MOF‐Derived MAMs via Texture Regulation

**Figure 2:**
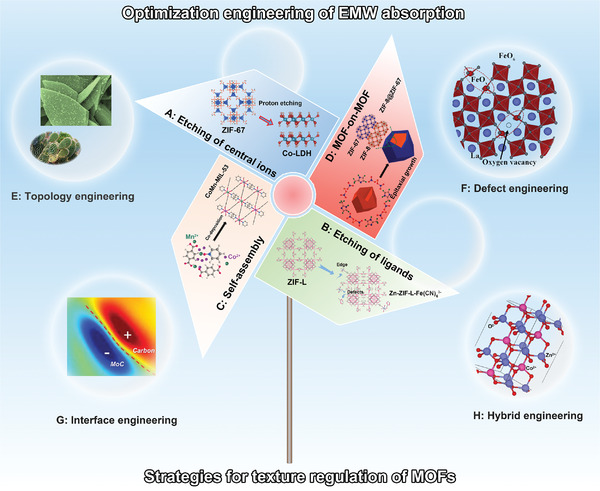
Schematic illustration of texture regulation strategies for the production of MOF derived MAMs, and the corresponding optimization engineering approaches for improved EMW absorption. The texture regulation strategies include a) etching and exchange of central ions. Reproduced with permission.^[^ ^44^ ^]^ Copyright 2021, Elsevier. b) Etching and exchange of ligands. Reproduced with permission.^[^ ^45^ ^]^ Copyright 2021, Elsevier. c) Chemically induced self‐assembly. Reproduced with permission.^[^ ^46^ ^]^ Copyright 2021, American Chemical Society. d) MOF‐on‐MOF heterostructure design. Reproduced with permission.^[^ ^47^ ^]^ Copyright 2020, Elsevier. The depicted optimization engineering approaches shows e) nano‐micro topology engineering. Reproduced with permission.^[^ ^31^ ^]^ Copyright 2019, American Chemical Society. f) Defect engineering. Reproduced with permission.^[^ ^48^ ^]^ Copyright 2021, Elsevier. g) Interface engineering. Reproduced with permission,^[^ ^49^ ^]^ Copyright 2022, Royal Society of Chemistry. h) Hybrid engineering. Reproduced with permission.^[^ ^50^ ^]^ Copyright 2021, Wiley‐VCH.

MOFs with diverse compositional and structural properties have been explored and validated as efficient MAMs.^[^ ^43^ ^]^ Fundamentally, the overall design concept of MOFs‐derived MAMs can be generalized into the following four stages: selection of coordination monomers (organic ligands and metal ions), controlling the topology via coordination‐driven self‐assembly, obtaining corresponding derivatives through postprocessing, checking EMW absorbability, and EM response mechanism. The EMW absorption of MOF derivatives is known to not only be determined by their intrinsic architectures and physical properties, but also be highly manipulated by the texture regulation, which affects the EM energy attenuation. Herein, we comprehensively summarized representative strategies for tuning EM properties and EMW absorption characteristics of MOFs via controllable textural regulation, such as etching and exchange of central ions, etching and exchange of ligands, chemically induced self‐assembly, and MOF‐on‐MOF heterostructure design (**Figure** **2**).

### 2.1 Etching and Exchange of Central Ions

Etching and exchange of central ions is a direct and highly effective strategy for modulating the texture of MOFs, which can lead to the evolution of aspects, such as chemical structure, crystal structure, and morphology. As summarized in **Table** **1**, central ions can be etched with three different etchant systems: protonic acids, transition metal ions, and both protonic acid and transition metal ions.

**Table 1:** Representative MAMs derived from MOFs with etching and exchange of central ions

MAMs	MOFs	Etchant	Texture regulation	*RL*_min_ [dB]	*f*_E_ [GHz]	Refs.
Co@NCNs	ZIF‐67	Protonic acid	Tannic acid etch ZIF‐67	−60.60	5.10	[51]
H‐MoC/NC	ZIF‐Zn		Tannic acid etch ZIF‐Zn	−41.20	5.20	[49]
CoNi/C	ZIF‐67	Transition metal ions	Ni^2+^ etch ZIF‐67	−61.80	10.2	[52]
CoNi/C‐PVDF	ZIF‐67		Ni^2+^ etch ZIF‐67	−61.02	5.20	[53]
Co_3_O_4_@NiCo_2_O_4_	ZIF‐67		Ni^2+^ etch ZIF‐67	−34.42	4.88	[54]
CoNi@C‐rGO	ZIF‐67		Ni^2+^ etch ZIF‐67	−58.20	4.03	[55]
Graphite/CoNi	ZIF‐67		Ni^2+^ etch ZIF‐67	−63.79	7.63	[56]
Co/NPC@ZnO/rGO	ZIF‐67		Zn^2+^ etch ZIF‐67	−45.40	5.40	[57]
Co‐LDHs/SCFs	ZIF‐67		Co^2+^ etch ZIF‐67	−40.40	6.50	[44]
LaCoO_3_/Co_3_O_4_	ZIF‐67		La^3+^ etch ZIF‐67	−45.91	6.88	[58]
Fe/C	ZIF‐8		Fe^2+^ etch ZIF‐67	−29.50	4.30	[59]
Co/Cu@C	Cu‐BTC		Co^2+^ etch Cu‐BTC	−52.50	5.68	[60]
Co@C@MnO	Co‐MOF‐74		Mn^2+^ etch Co‐MOF‐74	−64.40	6.70	[61]
CoFe@LaFeO_3_	CoFe‐PBA		La^3+^ etch CoFe‐PBA	−44.13	4.88	[48]
CoNi@BNC	ZIF‐67		Ni^2+^, H_3_BO_3_ etch ZIF‐67	−62.80	8.00	[62]

#### 2.1.1 Etching with Protonic Acids

**Figure 3:**
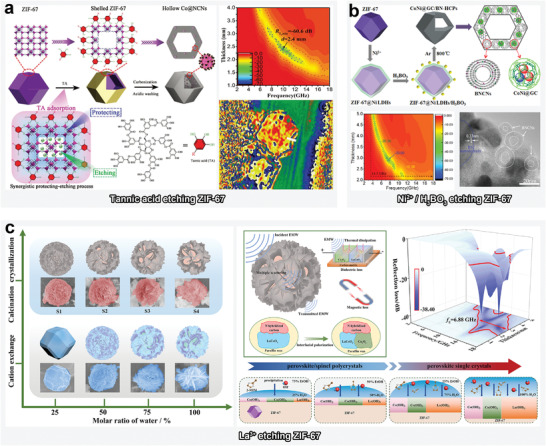
a) Schematic illustration of tannic acid‐based etching of ZIF‐67, the *RL* value, and the charge density map of as‐prepared hollow Co@NCNs‐800. Reproduced with permission.^[^ ^51^ ^]^ Copyright 2021, Wiley‐VCH. b) Schematic illustration of Ni^2+^/H_3_BO_3_‐based etching of ZIF‐67, the *RL* value, and high‐resolution transmission electron microscopy (HRTEM) image of as‐prepared CoNi@GC/BN‐HCPs. Reproduced with permission.^[^ ^62^ ^]^ Copyright 2019, American Chemical Society. c) Schematic illustration of La^3+^‐based etching of ZIF‐67, the *RL* value and the relative EMW absorption mechanism of as‐prepared LaCoO_3_/Co_3_O_4_. Reproduced with permission.^[^ ^58^ ^]^ Copyright 2021, Elsevier.

Typically, protonic acids rupture the coordination between metal ions and ligands, which leads to collapse of the MOF structure. Protonic acid etching is typically applied in the preparation of MOF‐precursor‐based lightweight MAMs with hollow and high‐porosity structures. Che et al. devised a synergistic protecting‐etching strategy to fabricate hollow ZIF‐67 polyhedrons and a derivative of hollow Co@N‐doped carbon nanocages (Co@NCNs) via rational introduction of tannic acid, which acted as both a protecting and etching species (**Figure** **3**a).^[^ ^51^ ^]^ ZIF‐Zn was also proven to be an ideal sacrificial template for hollow MAMs via tannic acid etching.^[^ ^49^ ^]^

#### 2.1.2 Etching with Transition Metal Ions

The transition metal ion etching of MOFs is the most popular mode of etching coordination center ions. This is based on the difference in the selectivity of coordination activity between metal ions and ligands. That is, the binding strength between the etchant and ligand must be greater than or close to the coordination strength between the metal ion of the original MOF and the ligand. Optimization of EMW absorption of metal‐ion‐etched MOF‐derived MAMs is typically performed using approaches, such as introducing new phases, constructing hybrid states and heterointerfaces, and regulating the microscopic topography. Moreover, the etching and exchange process is occasionally accompanied by the generation of new crystal phase.

For example, Co‐MOF‐74 can be etched with Mn^2+^ to yield pure MOF‐74 with perfect crystallinity without any impurity phases, which is referred to as a simulated MOF‐74 crystal.^[^ ^61^ ^]^ Ji et al. developed an adsorption calcination technique to etch Cu‐BTC with Co^2+^.^[^ ^60^ ^]^ By thoroughly grounding a mixture of Cu‐BTC and Co^2+^ in an ethanol solution, the Co^2+^ was absorbed into the pores and channels of the Cu‐BTC. After calcination, these MOFs transformed to carbon‐based composites, of which higher Co^2+^ etching concentration resulted in higher *ε*_r_ and tan*δ*_*ε*_ values.

In most cases, the etching of MOFs with metal ions induces change in the crystalline structure and even leads to the appearance of new phase. Gao et al. proposed a controllable La^3+^‐based ZIF‐67 etching strategy for N‐hybridized carbon‐based perovskite/spinel polycrystals (LaCo^3+^_1‐2_ *_*δ*_* Co^2+^_2_ *_*δ*_* O_3‐_ *_*δ*_*/Co_3_O_4_) with a tunable phase ratio, and valence state of Co ions and oxygen vacancy (V_O_) (Figure 3c).^[^ ^58^ ^]^ With increasing proportion of H_2_O, the protonation of Hmim gradually intensified, where the resulting OH^−^ generated by the splitting of H_2_O combined with Co^2+^ and La^3+^ to generate metal hydroxides, such as Co(OH)_2_ and La(OH)_3_. The as‐obtained MAMs inherited the characteristics of the MOF precursors as they exhibited phase transformation from perovskite/spinel polycrystals to perovskite single crystals. Finally, the polycrystal sample with moderate La^3+^ exchange showed stronger polarization relaxation, whose *f*_E_ value reached 6.88 GHz. Based on the aforementioned principles of etching ZIF‐67 with metal ions, numerous studies have been conducted on preparing hierarchical metal hydroxides, such as Co‐LDH,^[^ ^44^ ^]^ and CoNi‐LDH,^[^ ^54^ ^]^ thereby enhancing the number of MOF‐derived MAMs with diverse crystalline structures.

#### 2.1.3 Etching with Both Protonic Acid and Transition Metal Ions

Based on the advantages of introducing hollow structure via protonic acid etching and those of adjusting the crystalline structure via transition‐metal‐ion etching, a rational combination of these strategies has also been realized for the preparation of high‐performance lightweight MAMs with tunable component and structure. For instance, a simultaneous manipulation method has been proposed for modulating the chemical composition and microstructure of CoNi@graphitic‐carbon‐decorated B, N‐codoped hollow carbon polyhedrons derived from ZIF‐67 (Figure 3b).^[^ ^62^ ^]^ In this regards, Ni^2+^ was employed for etching the outer surface of ZIF‐67 to yield ZIF‐67@Ni LDHs, which were subjected to H_3_BO_3_ for etching the particles into the hollow structure.

### 2.2 Etching and Exchange of Ligands

In addition to etching central ions, the etching and exchange of ligands is another top‐down strategy for regulating the texture of MOFs; this includes both wet‐ and dry‐etching methods. According to the data summarized in **Table** **2**, the MOF derivatives that are subjected to etching and exchange of ligands mostly assist in yielding MAMs with hollow morphologies and new chemical components, especially metal sulfides.

**Table 2:** Representative MAMs derived from MOFs with etching and exchange of ligands

MAMs	MOFs	Etching type	Texture regulation	*RL*_min_ [dB]	*f*_E_ [GHz]	Refs.
Fe‐ZnO	ZIF‐L	Wet etching	Fe(CN)_6_^3−^ exchange Hmim	−33.22	4.24	[45]
Mo_2_N@CoFe@C/CNT	ZIF‐67		Fe(CN)_6_^3−^ exchange Hmim	−53.50	5.00	[63]
NiCo@C	NiCo‐PBA		Alkaline etch Co(CN)_6_^3−^	−68.40	5.80	[64]
Cu_31_S_16_	Cu‐MOF‐74		KOH/Na_2_S etch H_4_DOBDC	−15.10	6.20	[65]
Co‐C/Void/Co_9_S_8_	ZIF‐67		Thioacetamide etch Hmim	−54.02	8.20	[66]
Ag@C	Ag‐MOF‐5		Hmim etch H_2_BDC	−47.36	5.44	[67]
Cu/C	Cu‐BTC		(NH_4_)_2_MoS_4_ etch H_3_BTC	−52.00	6.80	[68]
Fe_7_S_8_/C	MIL‐88A	Dry etching	Thiourea etch MIL‐88A	−68.86	4.56	[69]
Air@Cu_2−_ *_x_* S@PI	HKUST‐1		S etch HKUST‐1	−40.60	X‐band	[70]
C/Cu/Cu_2_O/Cu_2−_ *_x_* S	Cu‐MOF‐74		S etch Cu‐MOF‐74	−33.50	7.60	[71]

#### 2.2.1 Wet Etching

Wet etching refers to the etching of suspended MOFs using an etchant dissolved in an etching solution. Generally, alkalis or ligands with strong binding ability to the central metal ions are used as etchants in this context, which result in hollow frameworks or new chemical states, respectively.

**Figure 4:**
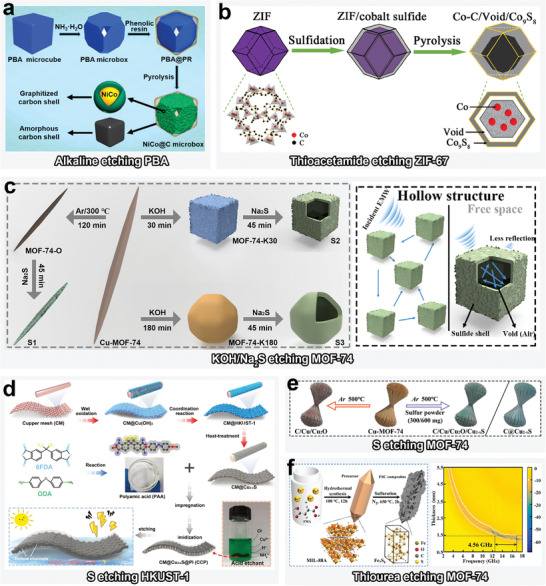
a) Schematic illustration of alkaline etching of PBA. Reproduced with permission.^[^ ^64^ ^]^ Copyright 2021, Elsevier. b) Schematic illustration of thioacetamide etching of ZIF‐67. Reproduced with permission.^[^ ^66^ ^]^ Copyright 2021, Springer Nature. c) Schematic illustration of KOH/Na_2_S etching of MOF‐74. Reproduced with permission.^[^ ^65^ ^]^ Copyright 2022, Elsevier. d) Schematic illustration of S etching of HKUST‐1. Reproduced with permission.^[^ ^70^ ^]^ Copyright 2021, Wiley‐VCH. e) Schematic illustration of S etching of MOF‐74. Reproduced with permission.^[^ ^71^ ^]^ Copyright 2022, Elsevier. f) Schematic illustration of thiourea etching MIL‐88A. Reproduced with permission.^[^ ^69^ ^]^ Copyright 2021, Elsevier.

In terms of alkaline etching, Du et al. developed a strategy for preparing hollow NiCo@C microboxes by etching Ni_3_[Co(CN)_6_]_2_·xH_2_O PBAs with an alkaline etchant—NH_3_·H_2_O in a H_2_O/ethanol mixed solution (**Figure** **4**a).^[^ ^64^ ^]^ The OH^−^ generated by the cleavage of HN_3_·H_2_O successfully converted PBA microcubes into PBA microboxes by stripping the inner Co(CN)_6_^3−^ ligands. With respect to assisted ligand etching, Wu et al. conducted a representative study on the controllable etching of the Hmim ligands in ZIF‐67 with Fe(CN)_6_^3−^, which included an exhaustive analysis of the principle of coordination chemistry during ligands exchange (Figure 2).^[^ ^45^ ^]^ Ligand exchange is also typically exploited in the preparation of transition metal sulfides, a class of semiconductor materials with excellent dielectric response. As shown in Figure 4b, a typical anion‐exchange reaction has been proposed to fabricate a Co–C/Co_9_S_8_ composite.^[^ ^66^ ^]^ In an ethanol solution, the mim^−^ component of ZIF‐67 was etched with thioacetamide, whereas yolk–shell‐structured Co–C/Void/Co_9_S_8_ ternary composite was obtained after calcination.

Given the advantages of the two aforementioned strategies, recent studies have started to pursue the preparation of MOF‐based hollow MAMs with tunable chemical structures through rational implementation of both alkaline etching and ligand exchange. As shown in Figure 4c, KOH and Na_2_S have been simultaneously employed in the etching of Cu–MOF‐74.^[^ ^18^ ^]^ In this regard, pseudomorphic transformation of MOF‐74 occurred under alkaline conditions, while the advancement of alkaline etching led to gradual evolution of the rod‐like topology into cubic (30 min) and dodecahedral forms (180 min). Hollow sulfides were subsequently obtained after these hard templates were etched with Na_2_S.

#### 2.2.2 Dry Etching

Dry etching is a method that requires high precision, in which the etching of MOFs is typically performed via vapor deposition. Almost all the MAMs prepared via dry etching have been used to synthesize transition metal sulfides. During the vapor deposition process, the upstream etchant is sublimated into the vapor phase at a high temperature and driven by the flow of inert gas. Then, it reacts with the downstream MOF precursor to generate sulfides of various valence states. Chemicals such as sulfur powder and thiourea are typically used as the sulfur source in dry etching.

Sulfur powders can be utilized to controllably etch HKUST (Hong Kong University of Science and Technology) MOF for synthesizing Cu_2−_ *_x_* S (Figure 4d),^[^ ^70^ ^]^ and to etch Cu–MOF‐74 for fabricating C/Cu/Cu_2_O, C/Cu/Cu_2_O/Cu_2−_ *_x_* S, and C@Cu_2−_ *_x_* S (Figure 4e).^[^ ^71^ ^]^ Thiourea is also considered as an ideal dry etchant for MOFs. For example, Liu et al. proposed a novel dry etching strategy to fabricate Fe_7_S_8_/C composites using a thiourea sulfuration process with MIL‐88A (Figure 4f).^[^ ^69^ ^]^ At temperature >400 °C, the H_2_S gas released via the decomposition of thiourea molecules acted as the actual etchant, which allowed S^2−^ to further chemically react with Fe^3+^.

### 2.3 Chemically Induced Self‐Assembly

Here, self‐assembly strategy refers to the controllable deposition of MOFs with different compositions and structures via chemical induction. As a bottom‐up synthesis approach, self‐assembly significantly enriches the texture of MOFs for EMW absorption. As summarized in **Table** **3**, the MOFs prepared by self‐assembly methods are generally designed using diverse metal ions, ligands, and topological regulation of supramolecular isomers (**Table** **4**).

**Table 3:** Representative MAMs derived from MOFs with chemically induced self‐assembly

MAMs	MOFs	Self‐assembly types	Texture regulation	*RL*_min_ [dB]	*f*_E_ [GHz]	Refs.
CoO/Zn*_x_* Co*_y_* O/ZnO	ZIF‐L	Diverse ions	Zn^2+^, Co^2+^ self‐assembly	−45.85	4.80	[50]
Co, Zn doped C	ZIF‐L		Zn^2+^, Co^2+^ self‐assembly	−45.20	5.70	[72]
Co/Co_3_ZnC	ZIF		Zn^2+^, Co^2+^ self‐assembly	−59.70	5.30	[73]
HGS@PAC	ZIF		Zn^2+^, Co^2+^ self‐assembly	−32.43	X‐band	[74]
Co@ZnO@C	ZIF		Zn^2+^, Co^2+^ self‐assembly	−61.90	5.50	[75]
Co@ZnO@C	ZIF		Zn^2+^, Co^2+^ self‐assembly	−52.60	5.80	[76]
CoFe@C	MOF‐74		Co^2+^, Fe^2+^ self‐assembly	−61.80	9.20	[77]
NiCo/C@CNT	MOF‐74		Co^2+^, Ni^2+^ self‐assembly	−58.80	6.50	[78]
FeCoNi@C	MOF‐74		Fe^2+^, Co^2+^, Ni^2+^ self‐assembly	−69.03	8.08	[79]
FeCo/MnO@NPC	MOF‐74		Fe^2+^, Co^2+^, Mn^2+^ self‐assembly	−54.07	7.72	[38]
Ni_1−_ *_x_* Co*_x_* @C	NiCo‐MOF		Co^2+^, Ni^2+^ self‐assembly	−39.30	4.80	[80]
ZnO‐Ni@CNT	NiZn‐MOF		N^i2+^, Zn^2+^ self‐assembly	−58.60	4.80	[81]
CoNi@NC/rGO	BTC		Co^2+^, Ni^2+^ self‐assembly	−68.00	6.70	[82]
Co/Ni/C	MOF‐71		Co^2+^, Ni^2+^ self‐assembly	−49.80	7.60	[30]
FeNi@CNT/CNRs	MIL‐88B		Fe^3+^, Ni^2+^ self‐assembly	−47.00	4.50	[34]
FeCo_2_@C	MIL‐88B		Fe^3+^, Ni^2+^/Co^2+^/Mn^2+^, self‐assembly	−71.40	14.2	[83]
Co/MnO/C	MIL‐53		Co^2+^, Mn^2+^ self‐assembly	−55.00	5.95	[46]
Fe*_x_* Ni_1−_ *_x_* @C	MIL‐100		Fe^2+^, Ni^2+^ self‐assembly	−71.30	5.30	[84]
Ni@C	Ni‐MOF	Diverse ligands	H_3_BTC, Hmim self‐assembly	−46.90	6.80	[85]
Ni/C	Ni‐MOF		H_3_BTC, pyrazine self‐assembly	−65.33	5.10	[86]
H‐MoC/NC	ZIF		MoO_4_^2−^, Hmim self‐assembly	−41.20	5.20	[49]
CoFe@C	PBA	Both diverse ions and ligands	Fe^3+^/Co^2+^, Fe(CN)_6_^4−^/Fe(CN)_6_^3−^ self‐assembly	−57.40	14.80	[87]
Fe/C	MIL‐101/ MIL‐88B	/	Supramolecular isomer	−59.20	6.50	[88]
Co@NC	ZIF‐67	/	Supramolecular isomer	−53.00	6.20	[89]

**Table 4:** Representative MAMs derived from MOF‐on‐MOF

MAMs	MOFs	Texture regulation	*RL*_min_ [dB]	*f*_E_ [GHz]	Refs.
ZnOC@CoC@PAN	ZIF‐8@ZIF‐67	Core–shell	−50.62	5.86	[95]
ZnO/NPC@Co/NPC	ZIF‐8@ZIF‐67		−28.80	4.20	[96]
Co/MnO/CNTs	ZIF‐8@ZIF‐67		−58.00	4.50	[97]
ZnO@C/Co_3_ZnC	ZIF‐8@ZIF‐67		−62.90	5.50	[98]
Co@C@NPC	ZIF‐8@ZIF‐67		−57.20	5.70	[99]
CoNi/TiO_2_	MIL‐125@ZIF‐67		−65.3	4.40	[100]
CoFe@C	ZIF‐67@PBA		−44.10	5.20	[101]
Co@ZnO/Ni@NC	ZIF‐67@ZIF‐8	Yolk–shell	−55.00	≈3.60	[102]
Co@NC	ZIF‐8@ZIF‐67	Hollow multishell	−52.50	4.40	[47]
Cu/NC@Co/NC	Cu‐HKUST@ZIF‐67	Core‐satellite	−54.13	5.19	[103]
CoFe/FeZr_2_/CoZr_2_/ZrO_2_	DUT‐52@MIL‐88B		−65.2	4.80	[104]
FeCoZn@C	MIL‐88B@MOF‐5		−53.10	6.00	[105]

#### 2.3.1 Diverse Ions

**Figure 5:**
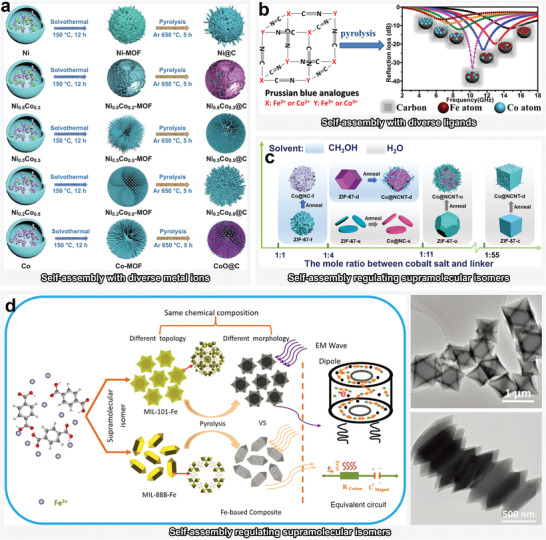
a) Schematic illustration of NiCo‐MOFs self‐assembly with tunable contents of Ni^2+^, Co^2+^. Reproduced with permission.^[^ ^80^ ^]^ Copyright 2021, Springer Nature. b) Schematic illustration of PBAs self‐assembly with tunable contents of Fe^2+^/Co^2+^ and K_4_Fe(CN)_6_·6H_2_O/K_3_Fe(CN)_6_/K_3_Co(CN)_6_. Reproduced with permission.^[^ ^87^ ^]^ Copyright 2018, Elsevier. c) Schematic illustration of self‐assembly‐regulated supramolecular isomers of ZIF‐67. Reproduced with permission.^[^ ^89^ ^]^ Copyright 2020, Wiley‐VCH. d) Schematic illustration of self‐assembly‐regulated supramolecular isomers of MIL‐101‐Fe and MIL‐88B‐Fe. Reproduced with permission,^[^ ^88^ ^]^ Copyright 2020, Wiley‐VCH.

Most of the self‐assembly strategies reported‐to‐date have focused on obtaining MOFs with diverse metal ions, such as Fe^2+^, Co^2+^, Ni^2+^, and Zn^2+^, which has enabled the preparation of multicomponent MAMs with modified heteroatoms and heterogeneous interfaces. Generally, the different metal ions used for codeposition must have similar ionic radii, electronic structure, electronegativity, and coordination ability with ligands.^[^ ^90^ ^]^ Otherwise, the one‐step self‐assembly fails, which can lead to phase separation and MOFs with subpar homogeneity. For example, H_4_DOBDC can be assembled with Fe^2+^, Co^2+^, and Ni^2+^ simultaneously for polymetallic MOF‐74;^[^ ^79^ ^]^ Zn^2+^ and Co^2+^ are typically used in bimetal ZIFs;^[^ ^73^ ^]^ H_2_BDC can be self‐assembled with Co^2+^, and Mn^2+^ into homogenous CoMn‐MIL‐53 (**Figure** **5**a).^[^ ^46^ ^]^

#### 2.3.2 Diverse Ligands

In general, the synthesis of MOFs via self‐assembly with diverse ligands is difficult because of the extremely harsh conditions required for homogeneous codeposition. Different ligands must have coordinating functional groups with similar electronic structures, consistent symmetry, and minimal coordination selectivity with metal ions. Otherwise, these ligands separately nucleate or coordinate with metal ions for step deposition. Generally, the long‐range order of PBAs family members is highly consistent; therefore, they can be used as an effective solution to adjust the chemical composition of magnetic alloys by regulating the ligands of PBAs (Figure 5b).^[^ ^87^ ^]^ Additionally, Che et al. confirmed that H_3_BDC and Hmim can be assembled simultaneously with Ni^2+^ to yield Ni‐MOFs.^[^ ^85^ ^]^ Ni^2+^ has also been simultaneously assembled with H_3_BDC and pyrazine to yield Ni‐MOFs for achieving a tunable morphology and EMW absorption.^[^ ^86^ ^]^

#### 2.3.3 Supramolecular Isomers

Several self‐assembly‐based strategies for the preparation of novel MOFs, such as the preparation of supramolecular isomers via chemical induction, have recently been developed. Generally, both the physical and chemical properties of MOFs can be considerably tuned owing to the diversity of the conformations, configurations, and topologies of supramolecular isomers, even if their chemical components are identical.^[^ ^91^ ^]^ Because the chemical structures of these MAMs are identical, their EMW absorption properties can assist in clarifying the dependence of EMW absorption on topography. For example, Chen et al. used two supramolecular MOF isomers (MIL‐101‐Fe and MIL‐88B‐Fe) as precursors for preparing Fe/C‐based EMW absorption composites (Figure 5d).^[^ ^88^ ^]^ Additionally, different polar solvents have been used for MOF self‐assembly to create supramolecular isomers (Figure 5c).^[^ ^89^ ^]^

### 2.4 MOF‐on‐MOF Heterostructures

Typically, MOF‐on‐MOF heterostructures are synthesized via epitaxial growth and stepwise deposition, with the former being an indirect template‐based method and the latter involving one‐pot reactions.^[^ ^92^ ^]^ The MOF‐on‐MOFs usually have structural features such as core/yolk–shell, hollow multishell, core–satellite, etc., and can therefore be adopted as ideal precursors for producing MAMs with heterogeneous interfaces.^[^ ^93^ ^]^ The structure of MOF‐on‐MOF materials is synergistically determined by the coordination selectivity of ligands and metal ions, the structural compatibility of the two MOFs, and the synthetic method.^[^ ^94^ ^]^

#### 2.4.1 Core/Yolk–Shell Heterostructure

Reasonable interfacial compatibility is a necessary condition for constructing MOF‐on‐MOF heterostructure; this typically depends on having an identical central ion or ligand.^[^ ^106^ ^]^ In other words, based on the nucleus of the original MOF crystals, MOF‐on‐MOF heterostructures with core–shell or yolk–shell configuration can be constructed through continuous epitaxial growth of different metal ions or ligands.

**Figure 6:**
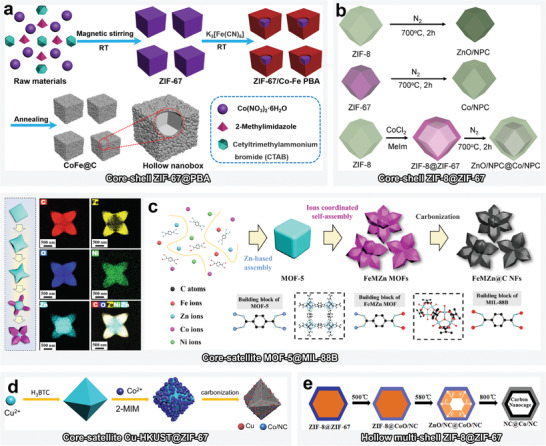
a) Schematic illustration for the preparation of core–shell‐type ZIF‐67@PBA and its derivative CoFe@C. Reproduced with permission, Copyright 2021,^[^ ^101^ ^]^ Elsevier. b) Schematic illustration for the preparation of core–shell ZIF‐8@ZIF‐67 and its derivatives ZnO/NPC, Co/NPC, and ZnO/NPC@Co/NPC. Reproduced with permission.^[^ ^96^ ^]^ Copyright 2016, Royal Society of Chemistry. c) Schematic illustration for the preparation of core–satellite MOF‐5@MIL‐88B and its derivative FeMZn@NFs. Reproduced with permission.^[^ ^105^ ^]^ Copyright 2022, Elsevier. d) Schematic illustration for the preparation of core–satellite Cu‐HKUST@ZIF‐67 and derivative Cu/NC@Co/NC. Reproduced with permission.^[^ ^103^ ^]^ Copyright 2022, Elsevier. e) Schematic illustration for the preparation of hollow multishell ZIF‐8@ZIF‐67 and derivative NC@Co/NC. Reproduced with permission.^[^ ^47^ ^]^ Copyright 2020, Elsevier.

In terms of the construction of MOF‐on‐MOFs with different ligands, Chen et al. used ZIP‐67 and CoFe‐PBA as the “core” and “shell,” respectively (**Figure** **6**a).^[^ ^101^ ^]^ To this end, ZIF‐67 nanoboxes were first assembled using Co^2+^ and Hmim. [Fe(CN)_6_]^3−^ was then epitaxially grown on the ZIF‐67 template as CoFe‐PBA by coordinating with Co^2+^ exchanged on the ZIF‐67 surface. MOF‐on‐MOF heterostructures prepared by epitaxial growth with identical ligands but different metals are remarkably popular, in particular, the Hmim‐based ZIF‐8@ZIF‐67 and ZIF‐67@ZIF‐8 (Figure 6b).^[^ ^96^ ^]^

#### 2.4.2 Hollow Multishell Heterostructure

Apart from the aforementioned template and etching methods for the preparation of hollow MAMs, manipulating the calcination process of MOF precursors can also yield hollow structure.^[^ ^107^ ^]^ A gradual increase in the pyrolysis temperature can enable the gas inside ZIF‐8@ZIF‐67 to induce an expansion force, which inflates the framework of the MOF‐on‐MOF into multilayer hollow shells (Figure 6e).^[^ ^47^ ^]^

#### 2.4.3 Core–Satellite Heterostructure

Homogeneous nucleation typically occurs during the synthesis of core–satellite structure, which is different from that in the two aforementioned, differently textured MOF‐on‐MOF configuration; that is, independent nucleation is not reliant on the nucleation and growth of the parent MOFs, whereas core–satellite structure nucleates on its own.^[^ ^108^ ^]^ However, core–shell structured MOF‐on‐MOF materials share the same core. Furthermore, both the template‐based epitaxial growth and one‐pot stepwise deposition strategies can be employed to synthesize core–satellite‐structured MOF‐on‐MOF materials.

Typically, the stepwise deposition strategy has been used to prepare core–satellite‐structured MOF‐5@MIL‐88B and derivative MAMs (FeMZn@C; M = Co or Ni) (Figure 6c).^[^ ^105^ ^]^ In this regard, owing to the active order of the coordination of H_2_BDC with Fe^3+^, Co^3+^, Zn^2+^, and Ni^2+^, the H_2_BDC first assembled with Zn^2+^ to form cubic MOF‐5 and then with Fe^3+^ to form shuttle‐like MIL‐88B, which determined the final nanoflower structure of the MOF‐on‐MOF. Epitaxial growth on a template is a more direct and efficient strategy for fabricating core–satellite MOF‐on‐MOF structures because the long‐range orders of both the MOFs can be ensured. For example, a series of core–satellite Cu‐HKUST@ZIF‐67 has been fabricated by in situ epitaxial growth of ZIF‐67 on Cu‐HKUST (Figure 6d).^[^ ^103^ ^]^

### 2.5 EMW Absorption Mechanism of MAMs Derived from MOFs via Texture Regulation

MAMs with tunable dielectric and magnetic properties can be obtained via the aforementioned texture regulation of MOFs. Generally, the EMW absorption performance can be calculated as follows^[^ ^109^ ^]^

(1) $$RL\;\left(\mathrm{dB}\right)=20\log\left|\frac{Z_{\mathrm{in}}-Z_{0}}{Z_{\mathrm{in}}+Z_{0}}\right|$$

(2) $$Z_{\mathrm{in}}=Z_{0}\sqrt{\frac{\mu_{r}}{\varepsilon_{r}}}\tanh\left(j\frac{2\pi fd}{c}\sqrt{\mu_{r}\varepsilon_{r}}\right)$$

where *ε*_r_ = *ε' – jε“* and *µ*_r_ = *µ' – jµ”* are the complex permittivity and complex permeability, respectively, whose real and imaginary parts represent storage and attenuation of dielectric and magnetic energy;^[^ ^110^ ^]^ *Z*_in_, *Z*_0_, *f*, *d*, and *c* are the input impedance of the MAMs, free space impedance, incident wave frequency, thickness of absorber, and light velocity, respectively;^[^ ^111^ ^]^ and *RL* is reflection loss (dB).When the *RL* value exceeds −10 dB, 90% of EMW can be absorbed, and the width of corresponding frequency band is defined as the effective absorption bandwidth (*f*_E_). Additionally, well‐matched impedance characteristics (|*Z*_in_/*Z*_0_|≈1) and high EM attenuation (*α*) are the basis for effective EMW absorption. Meanwhile, the EM energy attenuation is mainly attributed to dielectric and magnetic losses, which include conductive loss, polarization relaxation (such as dipole polarization and interfacial polarization), and the magnetic coupling effect. The basic EMW absorption mechanism of MAMs derived from MOFs via texture regulation is summarized below.

The etching and exchange of central metal ions can regulate EMW absorption of MOF‐derived MAMs not only by constructing hollow structures but also by introducing heterometal atoms or interfaces. First, the hollow structure induced by etching promotes the charge accumulation and distribution of active sites according to the charge‐density profile. Second, the exchange of metal ions can regulate the dielectric properties of final pyrolysis products. It also enables effectively introducing magnetic metal ions to nonmagnetic metal‐MOFs to improve magnetic loss, which consequently optimizes the impedance matching. Additionally, the modification of hetero‐metal atoms changes the crystal structure of the final MOF‐derived MAM products, and tunes the dielectric and magnetic properties.

The etching and exchange of ligands are typically performed to create heterogeneous structures and change the chemical state of metal derivatives, such as metal oxides and sulfides. The wet etching improves the specific surface area of MOF‐derived MAMs, which is remarkably beneficial in terms of multiple scattering and reflection. Exchanging the ligands constructs specific heterointerfaces, such as Schottky contacts, which can lead to interfacial polarization. The dry etching and exchange of ligands minimizes the loss of organic ligands, which enables the MOF derivatives to maintain a more complete carbon skeleton, and thereby ensuring that the MAMs have a strong conductive loss.

Chemically induced self‐assembly is predominantly used to develop hybrid MOFs and their derivatives (metal MAMs) with diverse chemical components and topologies. The self‐assembly facilitates control of metal‐ion composition and magnetic domain symmetry in magnetic alloys. It enhances magnetic dissipation and magnetic–dielectric synergy, resulting in high‐performance EMW absorption. Furthermore, heteroatoms and heterointerfaces can be constructed for further EMW absorption improvement.

MOF‐on‐MOF heterostructures efficiently integrate the advantages of various dielectric and magnetic materials and stimulate the synergistic dielectric and magnetic losses. As a result, absorption properties can be improved along with the broader effective absorption bandwidth. Additionally, heterointerfaces can be constructed in certain multiphase MAMs with rich morphological features.

## 3 Optimization Engineering of Texture Regulation of MOF‐Derived MAMs

MAMs with diverse structures can be derived from MOFs, as mentioned earlier in the texture regulation strategies. The mechanism by which the texture adjustment of MOFs affects the EM properties can be comprehensively explored by stablishing direct models to analyze the relationship between microstructure and EMW attenuation. A summary of the optimization engineering based on structural features, including nano‐micro topology engineering, defect engineering, interface engineering, and hybrid engineering, for EMW absorption has been provided (Figure 2). Furthermore, the EM response principles have been thoroughly reviewed based on individual material media; consequently, new insights into EMW absorption mechanisms are proposed.

### 3.1 Nano‐Micro Topology Engineering

The most intuitive and effective optimization engineering is the regulation of nano‐micro topology, which enables MOF‐derived materials with different physical and chemical properties to exhibit a specific structure that multiplies the EM response and EMW absorption with half of the effort. Generally, the forms of metal‐ions/ligands self‐assembly and MOF‐on‐MOF configuration considerably influence the nano‐micro topologies. Essentially, the nano‐micro topology engineering primarily involves the regulation of the morphology, porosity, size, and anisotropy of MOFs.

#### 3.1.1 Diverse Morphologies

Morphology is known to dramatically impact the dielectric and magnetic properties of MAMs, and even their EMW absorption performance. Certain studies have rationally selected ligands and metal ions to achieve control of the MOF dimensions through ingenious chemical induction methods. For example, Li et al. comprehensively investigated the relationship between the morphology and coordination modes of 3D rare‐earth MOFs via single‐crystal X‐ray diffraction.^[^ ^112^ ^]^ In particular, the difference in coordination between four types of metal ions (Y^3+^, Er^3+^, Yb^3+^, and La^3+^) and the oxygen and nitrogen atoms in maleic hydrazide ligands significantly altered the morphology of the MOFs, whereas the conjugated structure determined their electrical conductivity and high dielectric response activity. Results showed that the MOFs constructed with Er^3+^ have the highest *ε'* value. However, *ε“* values of all MOFs were almost similar between 1 and 2. Multidimensional MOFs exhibiting unique EM properties have also been developed. As shown in Figure 2, different molar ratios of Zn^2+^ and Co^2+^ have been used to synthesize 1D–2D multidimensional Co/N‐decorated carbon MAMs derived from CoZn‐ZIF‐L.^[^ ^31^ ^]^ After calcination, ZIF‐L pyrolyzed into composites with carbon nanotubes (1D) grafted on carbon flakes (2D), which was verified to be extremely effective for optimization of polarization loss and impedance matching. It is showed that the Co decorated carbon derived from ZIF‐L with Co^2+^/Zn^2+^ ratio of 1: 1 obtained the highest *ε”* value, indicating its best conductive loss.

**Figure 7:**
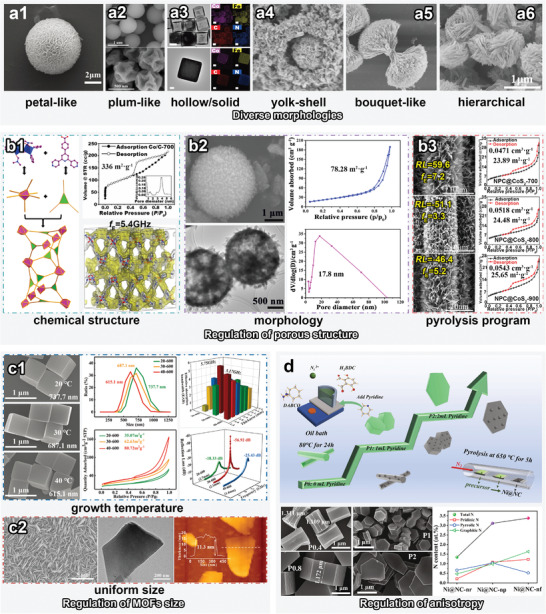
Schematic illustration of nano‐micro topology engineering. a1–a6) MOFs and related derivatives with diverse morphologies: a1) Petal‐like. Reproduced with permission.^[^ ^113^ ^]^ Copyright 2021, Royal Society of Chemistry. a2) Plum‐like. Reproduced with permission.^[^ ^114^ ^]^ Copyright 2022, Elsevier. a3) Hollow/solid. Reproduced with permission.^[^ ^115^ ^]^ Copyright 2020, Springer. a4) Yolk–shell‐like. Reproduced with permission.^[^ ^116^ ^]^ Copyright 2020, Elsevier. a5) Bouquet‐like. Reproduced with permission.^[^ ^71^ ^]^ Copyright 2022, Elsevier. a6) Hierarchical morphologies. Reproduced with permission.^[^ ^77^ ^]^ Copyright 2020, Elsevier. b1–b3) Regulation of porous structures based on b1) chemical structure. Reproduced with permission.^[^ ^117^ ^]^ Copyright 2019, American Chemical Society. b2) Morphology. Reproduced with permission.^[^ ^118^ ^]^ Copyright 2021, Springer. b3) Pyrolysis program. Reproduced with permission.^[^ ^119^ ^]^ Copyright 2020, Elsevier. c1) Regulation of MOFs size based on growth temperature. Reproduced with permission.^[^ ^120^ ^]^ Copyright 2021, Royal Society of Chemistry. c2) Images of MOFs with uniform size. Reproduced with permission.^[^ ^30^ ^]^ Copyright 2021, American Chemical Society. d) Regulation of anisotropy of MOFs. Reproduced with permission.^[^ ^126^ ^]^ Copyright 2022, Elsevier.

Hierarchical composites have also attracted growing attention owing to their complex polarization sites and coupled magnetic domains in particular geometries. A typical method for synthesizing hierarchical MOF‐74 is illustrated in **Figure** **7**a6.^[^ ^77^ ^]^ The morphologies of the MOFs were varied using tunable molar ratio of Co^2+^ and Fe^3+^ during the self‐assembly of Co^2+^/Fe^3+^ and H_4_DOBDC. The ɛ″ values decreased with the increase of Fe^3+^, indicating the lower dielectric loss of Fe nanoparticles. The as‐prepared hierarchically structured CoFe@C composites were confirmed to be characterized by lighter and wider‐band absorption (*f*_E_ = 9.20 GHz) thanks to multiple loss.

Various texture‐tuning strategies have yielded MOFs with remarkably different morphologies, which provide an avenue for investigating the morphological dependence of EMW absorption. Figure 7a1–a5 shows certain MOFs or their derivatives with typical morphologies, such as petal‐like Ni‐MOF,^[^ ^113^ ^]^ plum‐like NiCo@C,^[^ ^114^ ^]^ hollow/solid‐structured PBAs,^[^ ^115^ ^]^ yolk–shell‐type Ni@C@ZnO,^[^ ^116^ ^]^ and bouquet‐like MOF‐74.^[^ ^71^ ^]^ Typically, etching strategy leads to internal hollowing or surface modification of 3D materials; self‐assembly can be used to prepare materials with hierarchical structures and core–shell/yolk–shell structures; and MOF‐on‐MOF structures are also mainly used in core–shell materials (details in Section 2). For example, during the self‐assembly‐based synthesis of FeCo‐PBA and FeMn PBA cages, the simultaneous effect of trisodium citrate dihydrate and polyvinylpyrrolidone (PVP) induced the PBA to exhibit hollow structure (Figure 7a3). The results showed that the effective absorption bandwidth of MAMs with hollow structures were wider than those with solid structures, which presumably benefited from the multiple reflections and scattering in abundant cavities.

#### 3.1.2 Regulation of Porous Structure

Generally, MAMs with large pores facilitate multiple reflections and scattering, whereas large specific surface area produce interfacial polarization and consequently promote well‐balanced impedance matching. Porous MOFs also satisfy the design requirements of lightweight MAMs. Essentially, the porous structure of MOFs‐derived MAMs is determined by the chemical nature of MOFs, their unique morphology, and their calcination.

In terms of the chemical essence of MOFs, the structure of ligands is a critical factor which determines the 3D intercrossing cavities within the coordination polymer. For instance, 4’‐(4‐carboxyphenyl)‐4,2’:6’,4’’‐terpyridine (Hcptpy) has been employed to synthesize Co_2_O‐(cptpy)_2_(DMF) (CPT‐1‐Co) by coordinating with Co^2+^ (Figure 7b1).^[^ ^117^ ^]^ Owing to the large planar molecular structure and trifunctionality of Hcptpy, Brunauer–Emmett–Teller (BET) analysis indicated that CPT‐1‐Co exhibited an extremely high specific surface area (705.2 m^2^ g^−1^) and a predominant pore size of ≈14 Å, according to the calculations based on the density function theory (DFT). The specific surface area of the Co/C MAMs obtained by pyrolysis at 700 °C was as high as 336 m^2^ g^−1^, which led to a high *f*_E_ value of up to 5.40 GHz at a thickness of only 1.7 mm.

Certain unique morphologies significantly affect porous structures; MAMs with hollow, core–shell and yolk–shell structures tend to exhibit high porosity. The specific surface area and dominant pore size of the MOF‐derived hollow Ni/C microspheres prepared by Guo et al. were determined to be 78.28 m^2^ g^−1^ and 17.8 nm, respectively, by BET analysis (Figure 7b2).^[^ ^118^ ^]^ The hollow structure not only resulted in outstanding impedance matching, but also decreased the density of MAMs. Finally, the porous Ni/C samples calcinated at 600 and 700 °C showed typical dielectric relaxation type complex permittivity values, indicating the obvious polarization loss.

Calcination is also a noteworthy factor that affects the porous structure of MOF‐derived MAMs via modulating parameters such as the calcination temperature, time, and atmosphere. Generally, higher calcination temperatures result in higher porosities with numerous defects in the carbon networks, whereas excessively high temperatures lead to collapse of the framework, which consequently reduces porosity. A series of ZIF‐Ls was calcined at temperatures of 700, 800, and 900 °C (Figure 7b3).^[^ ^119^ ^]^ The specific surface areas of the samples calcined at 700, 800, and 900 °C were calculated to be 23.89, 24.48, and 25.65 m^2^ g^−1^, respectively; the corresponding dominant pore sizes were 0.0471, 0.0518, and 0.0543 cm^3^/g. It was concluded that the complex permittivity values increased with the specific surface areas, which can be attributed to the more surface polarization centers. The *RL*_min_ values of these specimens were −59.6, −51.1, and −46.4 dB, respectively.

#### 3.1.3 Regulation of MOF Size

Modifying the size of MOFs is indispensable to tuning the dielectric and magnetic losses. Smaller MOFs tend to be accompanied by more abundant grain boundaries and dipole centers, which are conducive to the construction of both electrically conducting networks and polarization loss sites. Additionally, magnetic losses generally rely on the stronger ferromagnetism of larger unit cells. Therefore, the preparation of MOF‐derived MAMs with controllable, uniform, and rational size distribution requires effective engineering strategy to realize balanced synergy between dielectric and magnetic losses.

Adjusting factors, such as the chemical deposition time and temperature, which alter the kinetics of the coordination assembly reaction, has been proven to be a highly effective approach for controlled regulation of the grain size of MOFs. For example, ZIF‐67 with adjustable average sizes has been synthesized by controlling the reaction temperature (20, 30, and 40 °C, Figure 7c1).^[^ ^120^ ^]^ Dynamic light scattering analysis showed that the particle size gradually decreased (737.7, 687.1, and 615.1 nm,) with increasing temperature, which may be attributed to the more significant increase in nucleation rate than that in grain growth. Additionally, the grain size reduction also led to an effective increase in the specific surface area, resulting in a significant increase in *ε*_r_.

Achieving uniform size control has long been considered a challenge in the synthesis of MOFs because their chemical environment constantly changes with respect to space and time. In particular, considerable attention has been devoted to overcoming the problems of agglomeration and dimensional nonuniformity in 2D MOFs. For instance, a versatile hydrothermal strategy has been employed to achieve confined growth of CoNi‐MOF‐71 nanosheet arrays with uniform thickness and lateral size (Figure 7c2).^[^ ^30^ ^]^ Results indicated that both the tan*δ_*ε*_* and tan*δ_µ_* values of 2D derivatives were higher than those of bulk derivatives of MOFs. The uniform ultrathin Co/Ni/C composite exhibited superior EMW absorption (*f*_E_ = 7.60 GHz) compared to that of bulk counterpart.

#### 3.1.4 Regulation of Anisotropy

Peculiar anisotropy‐related properties arise from the distinctive shape symmetries along different axes, compositional gradients, and even atomic arrangements.^[^ ^121^ ^]^ Generally, geometric anisotropy is expressed using three elements: arrangement, aspect ratio, and dimension faceting, which can be further evaluated based on orderliness (*O*), flatness (*F*), and heterotype (*H*), respectively. Furthermore, the anisotropy can be quantitatively evaluated based on geometric statistics according to the equation: *A* = *αHi* + *βFj* + *Ok*, where *A* is the assembly anisotropy vector; *α* and *β* devoted the correction factors of *H* and *F* with respect to *O*; and *i*, *j*, *k* are unit vectors in the three different dimensions, respectively.^[^ ^122^ ^]^ The anisotropy of MOFs affects both their dielectric and magnetic responses; however, the latter generally shows a stronger reaction. It has been reported that the high surface anisotropy of magnetic materials can attribute to high coercive force (*Hc*), which is beneficial to magnetic hysteresis loss, thus improving its EMW absorption.^[^ ^123^ ^]^ The synthesis of MOFs with controllable anisotropy has been attempted numerous times to achieve more efficient EMW absorption by modulating the chemical conditions for self‐assembly, such as mixing polar solvents and adding adjuvants.

For example, Wu et al. used different mass ratios of DMF and H_2_O to synthesize Co‐MOF‐71 with tunable anisotropy.^[^ ^124^ ^]^ By comprehensively comparing the *O*, *F*, and *H* values of the MOFs, the as‐obtained flower‐, boat‐, and bellow‐like products exhibited increasingly higher anisotropies owing to the induction of solvent proticity. Furthermore, simulation of the scattered EM fields and off‐axis electron holography revealed that the enhanced anisotropy led to a higher response frequency of natural resonance, a broader linewidth, and a superior magnetic loss.^[^ ^123^, ^125^ ^]^

Diversified adjuvants are considerably more popular in the preparation of anisotropic MOFs, such as auxiliary ions/ligands, inhibitors, and surfactants. For example, Zhang et al. found that the cationic surfactant cetyltrimethylammonium bromide (CTAB) increased the anisotropy of CoZn‐ZIF, which eventually took the shape of a six‐pointed star.^[^ ^75^ ^]^ It was determined that these “stars” with higher anisotropy showed higher tan*δ_*ε*_* but lower tan*δ_µ_* values, indicating that higher anisotropy of magnetic materials can lead lower dielectric loss but higher magnetic loss. The nonionic surfactant PVP was also confirmed to tune the anisotropy of a ZIF material by inducing preferentially oriented growth of the dominant crystal planes, thereby transforming grains into cuboids.^[^ ^76^ ^]^ The effects of pyridine molecules on the self‐assembly of H_2_BDC, 1,4‐diazabicyclo[2.2.2]octane, and Ni^2+^ were investigated (Figure 7d).^[^ ^126^ ^]^ As an inhibitor, each pyridine molecule provided only one nitrogen atom for connecting with Ni^2+^, which hindered the growth of hexagonal prisms along the *c*‐axis; therefore, the aspect ratio of the MOFs gradually decreased, resulting in hexagonal sheet‐like MOFs. Moreover, X‐ray photoelectron spectroscopy (XPS) analysis showed that the content of pyridine N in the Ni@NC composites gradually increased with pyridine content, which proved that pyridine played a role as surface‐capping polymer. Overall, the 2D layered structure with high specific surface area facilitated the construction of conductive networks, thus exhibiting a stronger conductive loss.

The anisotropic characters can modulate the EMW absorption performance by not only dielectric loss but also magnetic loss. On the one hand, high anisotropic materials, especially 2D dielectric materials have better surface conductivity and interfacial polarization. On the other hand, planar anisotropy materials are conductive to higher *µ*_r_ values ascribing to the easy magnetization planes where magnetic moments are preferably lying.^[^ ^127^ ^]^ In this case, we are supposed to take the deposition anisotropy of MOFs to prepare carbon composites with high surface anisotropy, of which the carbon components will be beneficial to the conductive loss. Most of the recent MAMs are dominated by dielectric loss, the MOFs derivatives with high orientation are also expected to be used to develop magnetic loss‐dominated MAMs with high magnetic permeability.

### 3.2 Defect Engineering

During the texture regulation of MOFs, numerous defects can be introduced to carbon‐based metal composites. Generally, the etching and exchange of central ions tend to break the long‐range order of the lattice, which promotes the formation of lattice defects, such as metal or oxygen vacancies, owing to the absence of certain metallic and nonmetallic atoms/ions. However, the etching and exchange of ligands is more effective in adjusting the defect structures in the carbon skeleton.

Defects affect the EMW response and absorption modes mainly by manipulating the dielectric properties of MAMs. Especially in semiconductor materials, defects may introduce complex charge carriers, including holes or electrons, and block or unblock corresponding charge‐migration channels, which play a crucial role in conductive loss. Additionally, localized defects can break the conservation of charge distribution by attracting or even trapping carriers, thereby inducing polarization relaxation. It is worth noting that defects‐induced polarization is different from classical dipole polarization, as defects can be classified into point (0D), line (1D), and planar defects (2D), whereas dipole polarization typically occurs on polar molecules or polar groups in materials.^[^ ^128^ ^]^ These planar defects may induce other forms of polarization, such as interfacial polarization at grain boundaries. In this regard, defect engineering can be analyzed based on the texture regulation of MOFs from three perspectives: vacancies on metal or oxygen sites in the metal‐containing lattices, and the defects in the carbon networks.

#### 3.2.1 Metal Vacancies

**Figure 8:**
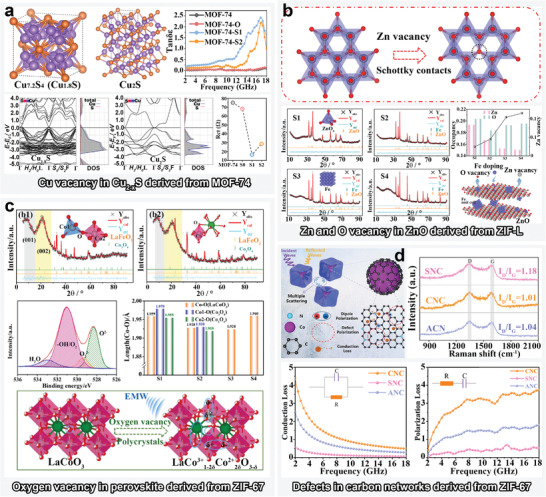
Schematic illustrations of the defect engineering schemes. a) Cu vacancy in Cu_2−_ *_x_* S derived from MOF‐74. Reproduced with permission, Copyright 2022,^[^ ^71^ ^]^ Elsevier. b) Zn and O vacancies in ZnO derived from ZIF‐L. Reproduced with permission.^[^ ^45^ ^]^ Copyright 2021, Elsevier. c) O vacancy in a perovskite derived from ZIF‐67. Reproduced with permission.^[^ ^58^ ^]^ Copyright 2021, Elsevier. d) Defects in carbon networks derived from ZIF‐67. Reproduced with permission.^[^ ^33^ ^]^ Copyright 2022, Elsevier.

In terms of lattice defects, metal and oxygen vacancies often exist simultaneously. In general, negligible amount of metal vacancies exist in the direct calcination products of MOFs precursors, because most MOFs have homogeneous long‐range order. According to the literature, this could be due to the fact that most metal vacancies in MOF derivatives are generally induced by texture regulation approaches, such as sulfide and heterogeneous‐metal‐ions modification. Metal sulfides have diverse crystalline structures due to their complex valence and coordination states, which provides an essential premise for the presence of metal vacancies in unit cells. For example, treating Cu‐MOF‐74 with different amounts of sulfur powders has yielded multiphase Cu_2−_ *_x_* S composites, in which the incompletely sulfide components contained abundant Cu vacancies.^[^ ^71^ ^]^ First‐principles DFT calculation of Cu_7.2_S_4_ and Cu_2_S was performed to clarify the effect of Cu_2−_ *_x_* S on the semiconductor or metallic performance (**Figure** **8**a). The band structure and density of states (DOS) analyses indicated that the samples with deep sulfuration state had smaller band and slightly larger electric conductivity. Electrochemical impedance spectroscopy (EIS) also confirmed this inference because the S1 and S2 in Figure 8b showed considerably lower values of charge transfer impedance (*R*_ct_), which was in well coincidence with the higher *ε* value. In terms of lattice defects induced by heterogeneous metal ions, Zhai et al. investigated the rational preparation of Co*_x_* S*_y_*/Ni*_x_* S*_y_* MAMs using Ni^2+^‐modified ZIF‐67.^[^ ^129^ ^]^ Vacancies on Co, Ni, and S sites in Co*_x_* S*_y_*/Ni*_x_* S*_y_* polycrystalline lattices were found to be stimulated by Ni^2+^‐based erosion of the overall texture of ZIF‐67, which led to an efficient multiple‐defects‐induced polarization, thereby boosting the effective EMW absorption on X and Ku bands.

#### 3.2.2 Oxygen Vacancies

With respect to oxygen vacancies, both metal ion and ligand exchange strategies have been found to decrease the occupancy of oxygen, thereby modifying the semiconductor properties to achieve high dielectric loss. In addition to classical oxygen vacancy analysis methods, such as XPS and photoluminescence (PL) spectroscopy, new Rietveld refinement techniques for X‐ray diffraction (XRD) patterns have also been explored for conducting more accurate semiquantitative analyses of the spatial distribution and concentration of oxygen vacancies in lattices.^[^ ^130^ ^]^ For example, Gao et al. found that perovskite‐metal‐oxide semiconductors, such as LaFeO_3_,^[^ ^48^ ^]^ LaCoO_3_,^[^ ^58^ ^]^ or LaNiO_3_,^[^ ^131^ ^]^ tend to form oxygen vacancies. As shown in Figure 2, the LaFeO_3_ perovskite derived from La^3+^ etching of PBA was found to have an oxygen vacancy on the O1(8*d*) site of FeO*_x_* by Rietveld refinement. Figure 8c provides more information regarding the oxygen vacancy in the perovskite derived from the La^3+^ etching of ZIF‐67 in terms of the determination, structural and polarization analyses. First, the Rietveld refinement was employed to obtain crystal‐related information by comparing the fitted XRD pattern with the observed data, whereas the XPS data were used to prove the accuracy of the preceding chemical structure analysis. Subsequently, the local stress concentration and distortion of the unit cells were inferred from data, such as the bond length and bond angle of characteristic ionic bonds as well as cell shrinkage or expansion, which are typically important factors in determining the polarity of materials. There were more obvious polarization peaks in the complex permittivity versus frequency curves. Furthermore, the as‐prepared LaCo^3+^_1‐2*δ*_Co^2+^_2*δ*_O_3‐*δ*_/Co_3_O_4_ polycrystal exhibited efficient Debye relaxation processes, as indicated by Cole–Cole semicircles, which helped the polarization loss in semiconductors with oxygen vacancy modification.

Figure 8b presents a typical strategy involving simultaneous manipulation of Zn and oxygen vacancies of ZnO derived from ligand‐exchanging ZIF‐L (ligand: Fe(CN)_6_^3−^) to achieve tunable EMW absorption.^[^ ^45^ ^]^ According to the Rietveld refinement analysis, the atomic occupancies of both Zn (2*b*) and O (2*b*) of the as‐prepared ZnO decreased with the introduction of Fe^3+^. Moreover, the calculated *c*/*a* values of all samples were smaller than that of ZnO with the perfect wurtzite structure (1.633), which further confirmed the presence of the Zn and O vacancies. Notably, lattice defects also caused shrinkage of the unit cells, ultimately leading to effectively elevated *ε*‐values, which implied a higher conductive loss and defect‐induced polarization loss.

#### 3.2.3 Defects in Carbon Networks

Numerous studies have attempted to explore the mechanism by which the effect of defects in MOF‐derived carbon networks influence their EMW absorption.^[^ ^132^ ^]^ Attributing to the porous characteristic of MOF precursors and the catalytic effect of metal ions during pyrolysis, a mass of defects such as edges, vacancies, heteroatom substitutions, and oxygen‐containing functional groups tend to appear in these calcined products.

Varying the calcination temperature is the most simple and effective method for regulating the defects in MOF‐derived carbon networks,^[^ ^133^ ^]^ although the texture regulation of MOFs also dramatically affects the carbon skeleton structures, especially via ligand etching and multiple‐ligand‐based self‐assembly (see Section 2). In terms of the temperature manipulation approach, the calcination temperature must be sufficiently high to form graphite nanocrystals; continuously elevated temperatures are known to result in defects.^[^ ^134^ ^]^ For the MOF precursor structure, in addition to the intrinsic chemical structure of MOFs, etching pretreatment is generally an effective means of creating random carbon frameworks and defects.

Defects in carbon matrices are crucial for the formation of local microcurrents and the asymmetric distribution of charges. In general, the defects in carbon networks can be examined based on the degree of graphitization from Raman spectra. As shown in Figure 8d, the defects in carbon derived from ZIF‐67 boxes were analyzed using Raman spectra. The two major modes at 1350 and 1580 cm^−1^ were attributed to the D and G bands; the former indicates disordered carbon and localized defects, whereas the latter indicates the first‐order scattering *E*_2g_ vibration mode of *sp* ^2^ bonds in graphite carbon skeletons.^[^ ^33^ ^]^ Additionally, the intensity ratio *I*_D_/*I*_G_ is typically used to evaluate the degree of graphitization and the defect conditions. A higher degree of graphitization of the carbon network was found to facilitate the construction of electron transport channels to achieve superior conductivity, whereas the presence of defects reduced dielectric losses but optimized impedance matching.

### 3.3 Interface Engineering

Interface engineering can enable the construction of novel carbon‐based polycrystals and consequently promote both dielectric and magnetic losses. In particular, excellent texture regulation of MOFs can be achieved through this strategy using novel heterointerface. Polycrystal MAMs with diverse heterointerfaces can be created by etching and exchange of central ions as well as self‐assembly, whereas different contact modes in MOF‐on‐MOFs can develop heterointerface‐type forms.

The intrinsic properties of each component material derived from MOFs are inherited, which determine the fundamental EM response properties of the composites. Additionally, the construction of heterointerfaces induces synergistic effects among components, such as interface polarization, spatial charge transport, band alignment, and pinning effect.^[^ ^135^ ^]^ Herein, the effects of heterointerface with certain representative structures on EMW absorption are summarized for carbon*–*carbon materials, carbon*–*metal derivatives, conductive metal*–*semiconductors, and interfaces between multiphase metal derivatives.

#### 3.3.1 Carbon–Carbon Interfacial Engineering

**Figure 9:**
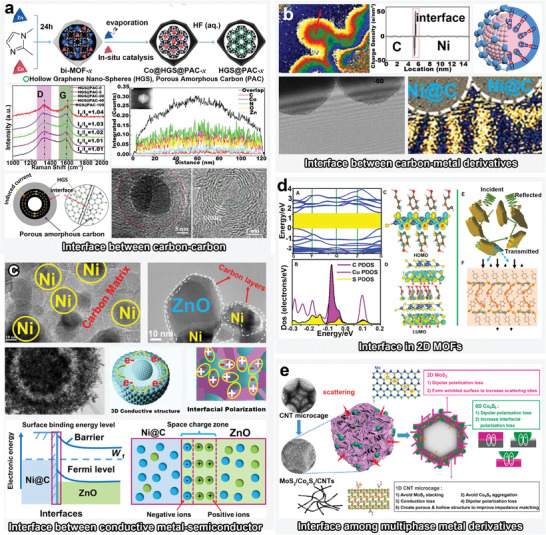
Schematic illustrations of the interface engineering strategies. a) Interface between graphene and amorphous carbon derived from ZnCo‐ZIF. Reproduced with permission.^[^ ^74^ ^]^ Copyright 2019, Elsevier. b) Interface between Ni and carbon derived from Ni‐MOF. Reproduced with permission.^[^ ^85^ ^]^ Copyright 2021, Elsevier. c) Interface between conductive metal Ni and semiconductive ZnO derived from NiZn‐MOF. Reproduced with permission.^[^ ^116^ ^]^ Copyright 2020, Elsevier. d) Interface in a 2D‐MOF. Reproduced with permission, Copyright 2022,^[^ ^137^ ^]^ Wiley‐VCH. e) Interfaces between multiphase metal derivatives. (Reproduced with permission.^[^ ^140^ ^]^ Copyright 2021, Elsevier.

Carbon materials acting as the matrix of MOF‐derived composites generally contain graphite and amorphous forms owing to the difference in crystallinity. Graphite has a considerable electrical conductivity owing to the efficient electron migration due to the large conjugated structural *sp* ^2^ networks, whereas amorphous carbon has a significantly lower electrical conductivity because of its defective structure. Nonetheless, the defects in amorphous carbon can create numerous polarization centers. In this scenario, spatial charge delocalization and even polarization relaxation occur at the heterointerface between graphite and amorphous carbon. As shown in **Figure** **9**a, a facile pyrolysis strategy has been devised to prepare bimetal ZnCo‐ZIF for yielding porous‐amorphous‐carbon‐coated hollow graphene nanospheres particles (HGS@PAC).^[^ ^74^ ^]^ Notably, a variable amount of Co was employed to controllably catalyze the graphitization of the MOFs in situ during pyrolysis, whereas HF‐based etching removed the metallic species from the composites. Consequently, the heterointerface ensured a significant conductive loss and interfacial polarization, prompting an effective absorption bandwidth that covered the entire X band.

#### 3.3.2 Carbon‐Metal Derivative Interfacial Engineering

The effects of the interface between the carbon matrix and metal derivative should be analyzed with respect to both dielectric and magnetic responses. The carbon matrix and metal derivatives, which have conducting and semiconducting properties, respectively, induce the accumulation of opposite charges in different phases and consequently promote energy decay. Additionally, ferromagnetic metal particles can also lead to a magnetic*–*dielectric synergistic effect to further optimize impedance matching. For example, Huang et al. directly observed the polarized charge distribution on the MoC(−)/carbon(+) interface for the first time by a hologram at the nanoscale.^[^ ^49^ ^]^ Through the self‐assembly of MoO_4_^2−^, Hmim, and Zn^2+^, MoC‐modified N‐doped hollow carbon spheres (H‐MoC/NC) were generated as the pyrolysis products. The charge density map in Figure 2 indicates that negative and positive charges are delocalized and aggregated on MoC and carbon materials, respectively, which was attributed to electrons excitation via Mo defects, thereby generating significant interfacial polarization during charge switching and accumulation. Additionally, as shown in Figure 9b, the effects of interface engineering on both dielectric and magnetic losses were simultaneously analyzed.^[^ ^85^ ^]^ The off‐axis electron holography and the charge density profile along selected regions suggested that charges concentrated on the heterointerface between Ni (positive) and carbon (negative), which facilitated the conductive loss and polarization relaxation behavior. Furthermore, the stray magnetic flux lines in reconstructed electron holography helped visualize the ferromagnetic responses for magnetic particles and conductive carbon, confirming the remarkable dielectric*–*magnetic compatibility and cooperation. Moreover, owing to the abundant types of MOFs with diverse ions and ligands (see Section 2.1.), the heterointerfaces between MOF‐derived carbon‐metal species were considerably enriched to optimize interface engineering for EMW absorption.

#### 3.3.3 Conductive Metal–Semiconductor Interfacial Engineering

The heterointerface constructed by conductive metals and semiconductors, which can be regarded as a Schottky contact, a type of special polarized interface, is worth discussing separately.^[^ ^136^ ^]^ The large difference in work function between metals and semiconductors leads to high interfacial resistance, thus leading to the bending of the semiconductor energy bands and the accumulation of space charges, which is also commonly referred to the Schottky barrier. The regional positive and negative charges can be driven to respond interfacial polarization under an alternating EM field for EMW attenuation. As shown in Figure 9c, Ni@C@ZnO microspheres with Schottky contact have been fabricated using NiZn‐MOF.^[^ ^116^ ^]^ The Ni@C and ZnO units worked as metalloid and semiconductor, respectively. Negative and positive charges concentrated on the conductive Ni@C and n‐type semiconductive ZnO, respectively, whereas the space charge zone facilitated intensive interfacial polarization. At a result, the Ni@C@ZnO absorbers with Schottky contact showed highest complex permittivity, whose the *RL*_min_ reached −55.8 dB. Furthermore, various MOF‐derived Schottky heterojunctions, such as Co‐MnO,^[^ ^61^ ^]^ Co‐ZrO_2_,^[^ ^19^ ^]^ and Fe‐ZnO,^[^ ^45^ ^]^ have been verified to be significantly effective for EMW absorption.

#### 3.3.4 Interfaces Between Multiphase Metal Derivatives

The heterointerface design of MOF‐derived polycrystalline MAMs has recently trended toward diversification and complexity for exploring their synergistic effects on the remarkable enhancement of EMW absorption. Studies are being conducted to comprehensively explore the mechanisms of the interfacial effects; moreover, achieving higher effective EMW absorption through rational manipulation of multicomponent heterointerfaces has also been a priority.

In terms of elucidating the mechanisms, DFT‐based simulations have also been performed in addition to the aforementioned testing methods such as off‐axis electron holography in recent studies on interface engineering. For instance, as shown in Figure 9d, the DFT results of a 2D MOF—CuHT (HT = 4‐hydroxythiophe)—indicated that highly dense pathways for charge transport can be generated on Cu–S layers.^[^ ^137^ ^]^ Moreover, the major EMW absorption mechanism in the 2D MOF semiconductor was confirmed to involve resistance loss by the conducting planes of the Cu_2_S layers, as the EM excitation promoted charges separation and charge‐carriers transport from Cu to aromatic rings.

In terms of multicomponent heterointerface, various interfaces have been randomly assembled using alloys, oxides, sulfides, carbides, and nitrides of metals.^[^ ^63^, ^68^, ^138^, ^139^ ^]^ As shown in Figure 9e, the heterointerfaces of MoS_2_‐Co_9_S_8_, MoS_2_‐CNTs, and Co_9_S_8_‐CNTs have been constructed in the 0D‐1D‐2D hybridized Co_9_S_8_/CNTs/MoS_2_ composite derived from ZIF‐67, among which the synergistic polarization loss boosted the *f*_E_ up to 8.4 GHz.^[^ ^140^ ^]^

### 3.4 Hybrid Engineering

Hybrid engineering of MAMs based on the texture regulation of MOFs is becoming a new research hotspot. The aforementioned four texture regulation strategies significantly affect the hybrid state of MAMs. The contribution of hybrid engineering to EMW absorption involves both the development of MAMs with unique structures and the exploration of new EMW absorption mechanisms. Numerous advanced MOF‐derived MAMs with diverse hybrid forms have been prepared by introducing new chemical components or adjusting the synthesis process, including heteroatoms‐doped carbon networks, heteroatoms‐doped semiconductors, ion and phase hybridization, as well as single atom hybridization. Additionally, several new EMW absorption mechanisms or analytical methods have been proposed, such as magnetoelectric coupling, balance of impedance matching and EM attenuation, and the synergetic polarization effect.

#### 3.4.1 Advanced MOF‐Derived MAMs with Diverse Hybrid Forms

##### Heteroatoms‐Doped Carbon Networks

**Figure 10:**
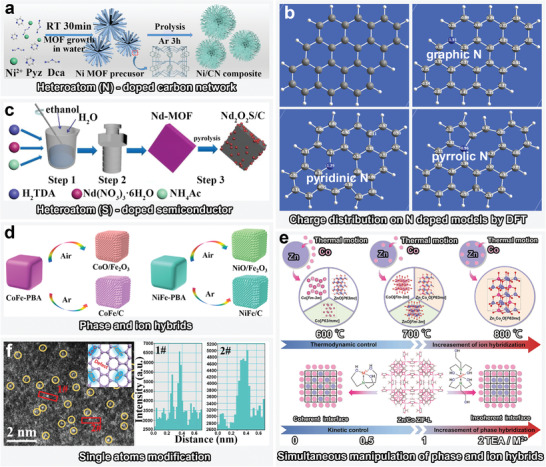
Schematic illustration of hybrid engineering approaches. a) Fabrication of N‐doped carbon derived from Ni‐MOF. Reproduced with permission.^[^ ^143^ ^]^ Copyright 2021, Elsevier. b) DFT‐based simulation of charge distribution on N‐doped models. Reproduced with permission.^[^ ^74^ ^]^ Copyright 2019, Elsevier. c) Fabrication of S‐doped Nd_2_O_2_S semiconductor derived from Nd‐MOF. Reproduced with permission.^[^ ^144^ ^]^ Copyright 2021, Wiley‐VCH. d) Pyrolysis‐atmosphere‐controlled ion and phase hybridization of PBA derivatives. Reproduced with permission.^[^ ^145^ ^]^ Copyright 2021, Elsevier. e) Simultaneous manipulation of Zn/Co phase and ion hybrids derived from ZnCo‐ZIF‐L. Reproduced with permission.^[^ ^50^ ^]^ Copyright 2021, Wiley‐VCH. f) Single‐Zn‐atom‐anchored N‐doped carbon derived from ZIF‐8. Reproduced with permission.^[^ ^146^ ^]^ Copyright 2021, Wiley‐VCH.

As introduced above, the MOF‐derived carbon networks play a significant role in the optimization of dielectric loss. Therefore, investigating the effects of heteroatoms (N^[^ ^141^ ^]^ and S,^[^ ^142^ ^]^ for instance) on carbon networks is critical. Almost all heteroatom‐doped MOF‐derived carbon materials have been realized by introducing heteroatom‐containing organic ligands via an etching or self‐assembly strategy. For example, as shown in **Figure** **10**a, the N‐containing pyrazine has been used as the organic ligand for self‐assembly with sodium dicyanamide and Ni^2+^ to yield Ni‐MOF and the corresponding N‐doped carbon composite.^[^ ^143^ ^]^ The doped N heteroatoms were regarded as polarization centers for inducing dipole polarization. With respect to the mechanism by which heteroatom doping regulates the charge properties, the charge distribution has been computationally investigated. As shown in Figure 10b, DFT calculations indicated that the charges on graphic‐, pyridinic‐, and pyrrolic‐N‐doped C_30_H_14_ models were −1.35, −1.29, and −0.96 eV, respectively, whereas all the surrounding C atoms were positive charged.^[^ ^74^ ^]^ Therefore, the N dopant was found to separate positive and negative charges, and consequently attenuate the EM energy by dipole polarization.

##### Heteroatoms‐Doped Semiconductors

Except for intrinsic semiconductors, the increase in charge‐carrier concentration of most semiconductors is attributed to defects or heteroatoms doping. Especially for certain metal oxide semiconductors, oxygen vacancies can often play the role of donors, thus exhibiting n‐type electronic conductivity. Rational chemical structural tuning of MOFs can be performed to prepare heteroatom‐doped semiconductors. As shown in Figure 10c, the Nd_2_O_2_S semiconductor was prepared using Nd‐MOF with a S‐containing ligand (2,5‐thiophenedicarboxylic acid).^[^ ^144^ ^]^ The conductivity of Nd_2_O_3_, a typical rare‐earth‐metal‐oxide p‐type semiconductor, was dramatically improved by substituting an O atom by a S atom. Simultaneously, the S occupancy on the O sites also induced abundant defects, which further enhanced the polarization loss. Finally, the *f*_E_ and *RL*_min_ values reached 14.48 GHz and −52.3 dB.

##### Ion and Phase Hybridization

Most derivatives of bimetallic and even multimetallic MOFs exhibit ion or phase hybrid structures. Ion hybridization refers to the existence of two metals in identical phases as a solid solution, whereas phase hybridization refers to the existence of two metals in two separate phases. Moreover, the hybrid forms of these metal‐based MAMs have been confirmed to significantly influence the dielectric and magnetic properties of the materials. Typically, the hybrid form of MOF‐derived multimetallic materials is not only determined by the chemical nature of the MOFs, but is also affected by the texture regulation and calcination conditions (atmosphere and temperature). Controllable texture regulation can be effectively achieved by ion exchange,^[^ ^60^ ^]^ ion adsorption,^[^ ^19^ ^]^ ligand etching, and self‐assembly codeposition. With respect to the calcination conditions, the atmosphere and calcination temperature are the main influencing factors. As shown in Figure 10d, Ji et al. investigated the effects of the calcination atmosphere on the hybridization form of PBA‐derived CoFe and NiFe hybrids.^[^ ^145^ ^]^ The pyrolysis products of CoFe‐PBA and NiFe‐PBA obtained in air exhibited phase separated metal oxide states (CoO/Fe_2_O_3_, NiO/Fe_2_O_3_), whereas those in an Ar atmosphere showed a metal alloy structure (CoFe, NiFe). Both the CoFe and NiFe alloys exhibited higher complex permittivity values than phase‐separated metal oxide samples. However, ionic hybridization and phase hybridization often coexist in MOF‐derived metallic MAMs. In this regard, researchers have manipulated ion and phase hybridization simultaneously through kinetic and thermodynamic control strategies (Figure 10e).^[^ ^50^ ^]^ In terms of kinetics, the difference in complexation constants between Zn^2+^ and Co^2+^ hydrometallatranes (Zn^2+^ > Co^2+^) dominated the stepwise Zn/Co deposition into ZIF‐L precursors. With respect to thermodynamics, the gradient in pyrolysis temperature yielded controllable ion hybridization products owing to different thermal motions.

##### Single‐Atom Hybridization

Certain MOFs have recently been used to prepare MAMs with novel hybrid forms using approaches, such as single‐atom modification. Single atoms with high activity enable the modification of electronic states of the metal element. For example, a series of single‐Zn‐atom‐anchored carbon layers was synthesized using ZIF‐8.^[^ ^146^ ^]^ The high‐angle annular dark‐field scanning electron microscopy (HAADF‐STEM) image and intensity profiles (Figure 10f) indicated that the isolated bright dots that were clearly distinguished from the heteroatom‐doped carbon matrix were single Zn atoms. Abundant Zn–C and Zn–N dipoles were found to be constructed by the Zn single atoms, which considerably boosted the dipole‐moment‐related dissipation. To clarify the polarization effects of hetero‐substitutions on EMW absorption, Zhang et al. analyzed the EMW absorption performance of single Fe atoms, subnanometer Fe clusters, and Fe_3_O_4_‐nanoparticle‐decorated nitrogen‐doped carbon nanocage derived from FePc@ZIF‐8, respectively.^[^ ^147^ ^]^ It can be concluded based on this work that single atoms samples showed higher complex permittivity values and lower complex permeability, attributing to abundant polarization sites, but it is difficult to have strong magnetic domains to form.

#### 3.4.2 Mechanisms for EMW Absorption Optimization of Hybrid Materials

In general, the hybrid engineering of MAMs derived from MOFs with texture regulation has been proposed to elucidate several novel EMW dissipation mechanisms. Tunable hybrid structures of carbon materials and metal derivatives can be obtained by modulating organic ligands and metal ions, which affects the properties of conductive networks, semiconductors, or magnetic particles, respectively. Overall, the various aforementioned hybrid forms indicate that the heteroatoms doping can create abundant polarization sites for polarization loss, whereas the formation of certain hybrid phases can not only alter the dielectric and magnetic properties, but also induce interface polarization. Furthermore, certain simultaneous or synergistic EMW absorption modes can be excited by these complex hybrid structures.

Overall, the hybridization of dielectric and magnetic materials is conducive to impedance matching. For instance, the distribution of the confined components of Zn/Co in Co@NC‐ZnO derived from ZnCo hybrid MOFs has been designed to achieve magnetic–dielectric balance.^[^ ^148^ ^]^ Essentially, the tunable ratio of Zn^2+^ to Co^2+^ determined the amount of the semiconductor ZnO and magnetic Co particles, which resulted in an efficient magnetoelectric coupling and well‐matched impedance. Furthermore, the aforementioned ion hybridization and phase hybridization were confirmed to favor defect polarization and dipole polarization, respectively, whereas their simultaneous contribution promoted a powerful synergistic polarization loss effect.^[^ ^50^ ^]^

## 4 Summary and Perspectives

**Figure 11:**
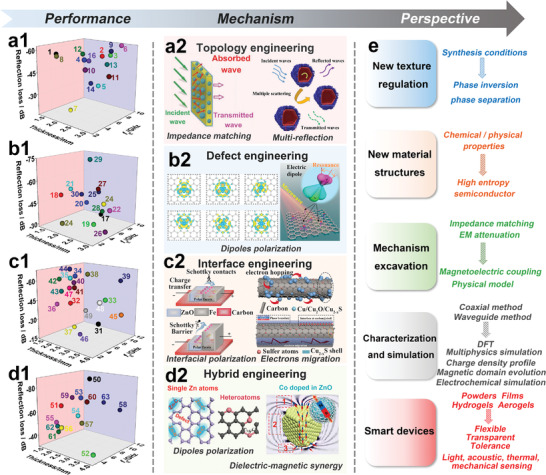
A comprehensive summary of EMW absorption performance (with respect to *RL*_min_, *f*_E_, and thickness) of representative MAMs optimized by a1) nano‐micro topology engineering, b1) defect engineering, c1) interface engineering, and d1) hybrid engineering. Primary EMW absorption mechanisms in a2) nano‐micro topology engineering. Reproduced with permission.^[^ ^80^ ^]^ Copyright 2020, Springer. Reproduced with permission.^[^ ^149^ ^]^ Copyright 2022, Elsevier. b2) Defect engineering. Reproduced with permission.^[^ ^15^ ^]^ Copyright 2021, Elsevier. c2) Interface engineering. Reproduced with permission.^[^ ^45^ ^]^ Copyright 2021, Elsevier. Reproduced with permission.^[^ ^71^ ^]^ Copyright 2022, Elsevier. d2) Hybrid engineering. Reproduced with permission^[^ ^146^ ^]^ Copyright 2021, Wiley‐VCH. Reproduced with permission.^[^ ^71^ ^]^ Copyright 2022, Elsevier. Reproduced with permission.^[^ ^148^ ^]^ Copyright 2021, Wiley‐VCH. e) A perspective for MOFs derivative MAMs.

Texture regulation of MOFs has been regarded as a highly promising strategy for preparing MAMs with unique components and configurations to achieve tunable EM responses. In this review, texture regulation strategies, such as etching and exchange of central ions, etching and exchange of ligands, chemically induced self‐assembly, and MOF‐on‐MOF heterostructure design, were systematically reviewed. More importantly, crucial insights on texture regulation have been provided, specially with respect to the EMW absorption mechanisms based on structural optimization engineering of MOF‐derived MAMs (**Figure** **11** and **Table** **5**). Particularly, the etching and exchange of central ions or ligands were discussed as remarkably effective for constructing hollow structures as well as introducing heterometal atoms and defects. Chemically induced self‐assembly mainly optimizes the hybrid states of anionic/cationic doping and heterogeneous multiphase MAMs. MOF‐on‐MOF heterostructures primarily contribute to investigations of the influence of heterointerfaces on EMW absorption, in particular interfacial polarization. Essentially, the texture regulation provides enormous opportunities and broad prospects for MAMs with respect to investigations of their EMW absorption mechanisms, human health, national defense security, as well as unmanned transportation systems.

**Table 5:** A comprehensive summary of EMW absorption performance of representative MAMs optimized by nano‐micro topology engineering, defect engineering, interface engineering, and hybrid engineering

No.	MAMs	Optimization engineering	Structure	Thickness [mm]	*f*_E_ [GHz]	*RL*_min_ [dB]	Refs.
1	Ni@C	Nano‐micro topology	Petal‐like	1.4	4.39	−55.99	[113]
2	NiCo@C		Plum‐like	2.5	7.2	−55.4	[114]
3	Fe/Co/C		Hollow/solid	2.5	8.8	−54.6	[115]
4	Ni@C@ZnO		Yolk–shell	2.5	4.1	−55.8	[116]
5	Cu_2−_ *_x_* S/Cu_2_O/Cu		Bouquet‐like	2.3	7.6	−33.5	[71]
6	CoFe@C		Hierachical	2.8	9.2	−61.8	[77]
7	Co/C		Chemical‐porous structure	1.7	5.4	−15.7	[117]
8	Ni@C		Morphology‐porous structure	1.56	3.8	−55.4	[118]
9	CoS_2_@C		Prolysis‐porous structure (700 °C)	2.8	7.2	−59.6	[119]
10	CoS_2_@C		Prolysis‐porous structure (800 °C)	2.7	3.3	−51.1	[119]
11	CoS_2_@C		Prolysis‐porous structure (900 °C)	3.1	5.2	−46.4	[119]
12	Co/NC		Growth temperature‐MOFs size	2.1	5.75	−56.92	[120]
13	Co/Ni/C		Distribution‐MOFs size	2.6	7.6	−49.8	[30]
14	Ni@NC		Anisotropy (flakes)	2.3	6.21	−37.11	[126]
15	Ni@NC		Anistoropy (particles)	7.2	6.25	−48.84	[126]
16	Ni@NC		Anisotropy (rods)	2.3	5.85	−52.88	[126]
17	Cu_2−_ *_x_* S/Cu_2_O/Cu	Defects	Metal vacancy (Cu)	2.3	7.6	−33.5	[71]
18	Co*_x_* S*_y_*/Ni*_x_* S*_y_*		Metal vacancy (Co, Ni)	1.5	3.95	−48.3	[129]
19	Fe‐ZnO		Metal and O vacancies (Zn)	2.6	4.24	−33.22	[45]
20	NiCo/CeO_2_/Ti_3_C_2_T*_x_*		O vacancy	2.0	6.32	−42.48	[150]
21	CeO_2−_ *_x_*/RGO		O vacancy	1.5	5.84	−50.6	[151]
22	CoFe/LaFeO_3_/La_2_O_3_		O vacancy	3.0	4.88	−44.13	[48]
23	LaCoO_3_/Co_3_O_4_		O vacancy	2.7	5.6	−45.91	[58]
24	Ni/NiO@C		Defects in carbon	2.0	1.65	−34.13	[132]
25	HCF@NC/Co		Defects in carbon	2.25	7.36	−50.14	[133]
26	Co/TiO_2_‐C		Defects in carbon	3.0	4.04	−31.0	[152]
27	CNT/Ni@N‐C		Defects in carbon	2.5	5.7	−55.1	[153]
28	Co_3_O_4_@C@*α*‐Fe_2_O_3_		Defects in carbon	2.5	6.6	−38.4	[154]
29	Ni/C/porous carbon		Defects in carbon	2.2	5.8	−73.8	[155]
30	Co@C		Defects in carbon	1.88	5.97	−48.3	[33]
31	HGS@PAC	Interface	Carbon‐carbon	3.70	4.2	−32.43	[74]
32	H‐MoC/NC		Carbon‐metal derivatives	2.0	5.2	−41.2	[49]
33	Ni@C		Carbon‐metal derivatives	3.5	6.8	−46.9	[85]
34	ZnO/C/Co_3_ZnC		Carbon‐metal derivatives	2.2	5.5	−62.9	[98]
35	CoNi/C		Carbon‐metal derivatives	2.0	5.2	−61.02	[53]
36	Fe/Fe_3_O_4_/C		Carbon‐metal derivatives	1.4	4.44	−39.2	[156]
37	Co_3_O_4_/C		Carbon‐metal derivatives	2.0	6.08	−20.3	[157]
38	Co@C@MnO		Conductive metal‐semiconductor	2.6	6.7	−64.4	[61]
39	Co/ZrO_2_/C		Conductive metal‐semiconductor	3.3	11.9	−57.2	[19]
40	ZnO‐Ni@C		Conductive metal‐semiconductor	2.3	4.8	−58.6	[81]
41	Ni@C@ZnO		Conductive metal‐semiconductor	2.5	4.1	−55.8	[116]
42	Co/MnO/CNTs		Conductive metal‐semiconductor	1.32	5.36	−58.6	[97]
43	CuHT		Multiphase metal derivatives	1.74	4.2	−50.9	[137]
44	Co_3_ZnC‐Co@N/C		Multiphase metal derivatives	1.97	5.23	−67.97	[139]
45	Co_9_S_8_/CNTs/MoS_2_		Multiphase metal derivatives	4.0	8.4	−35.4	[140]
46	Cu_2_S‐Cu_31_S_16_		Multiphase metal derivatives	2.3	6.2	−15.1	[65]
47	Mo_2_N@CoFe@C/CNT		Multiphase metal derivatives	2.0	5.0	−53.5	[63]
48	SiC/Ni/NiO/C		Multiphase metal derivatives	4.0	2.96	−50.52	[138]
49	Cu_2−_ *_x_* S/Cu_2_O/Cu		Multiphase metal derivatives	2.3	7.6	−33.5	[71]
50	Ni@N‐doped C	Hybrid	Heteroatoms (N) doped C	2.7	7.4	−86.8	[141]
51	Ni@N‐doped C		Heteroatoms (N) doped C	1.9	4.6	−65.0	[143]
52	HGS@PAC		Heteroatoms (N) doped C	3.70	4.2	−32.43	[74]
53	Co@S‐doped C		Heteroatoms (S) doped C	2.6	6.0	−72.3	[142]
54	S/Co@C		Heteroatoms (S) doped C	2.2	6.88	−54.5	[158]
55	TiO_2_/C		Heteroatoms (O) doped C	1.6	4.6	−49.6	[159]
56	Nd_2_O_2_S/C		Heteroatoms (S) doped Semiconductors	2.56	2.4	−52.3	[144]
57	Cu/Co/C		Phase hybrids	2.8	5.44	−52.5	[60]
58	Co/ZrO_2_/C		Phase hybrids	3.3	11.9	−57.2	[19]
59	Co@NC‐ZnO		Phase hybrids	1.9	6.8	−69.6	[148]
60	Fe*_x_* Ni_1−_ *_x_* @C		Ions hybrids	3.1	5.3	−71.3	[84]
61	NiFe/C		Phase and ion hybrids	1.65	4.8	−41.0	[145]
62	ZnO/Zn*_x_* Co*_y_* O/CoO		Phase and ion hybrids	1.6	4.8	−45.85	[50]
63	Fe@NCNs‐8		Single atoms hybrids	2.8	6.6	−26.18	[147]
64	Fe_3_O_4_@Zn‐N‐C		Single atoms hybrids	2.5	11.5	−61.9	[146]

The aforementioned summary suggests that research on the EMW absorption induced by the texture regulation of MOFs remains in its infancy. Therefore, numerous challenges must be overcome to prepare efficient MOF‐derived MAMs and construct corresponding physical models. The following perspectives could be of assistance in this regard:

1. Novel strategies for texture regulation. The most fundamental challenge involves achieving precise and controllable texture regulation of MOFs and developing novel implementation techniques. Conventional strategies including etching, self‐assembly, and MOF‐on‐MOF structural design are primarily based on manipulating factors, such as reaction temperature, pressure, pH, solvent, and surfactant. Thus, new control methods, cross‐fusion, or simultaneous implementation of these control methods could considerably broaden the types of texture regulation strategies.
2. Diverse material structures. The structure of MOF derivatives determines the EM response properties of MAMs; therefore, the development of MAMs with new structural features continues to show great promise. Topology has been found significantly influence both the dielectric and magnetic properties. However, the MAMs derived from multidimensional MOFs have not been systematically studied for EMW absorption, which may be conductive to the multiple attenuation and broad band absorption. Additionally, although the effect of size on EMW absorption has been validated, studies on size distribution are rarely reported. In terms of defect engineering and interface engineering, both the classification and quantification aspects require further exploration. Hybrid engineering is an extremely promising research direction because numerous active sites that facilitate EMW uptake can be created. For example, certain novel hybrid materials may exhibit unexpected properties, such as high‐entropy semiconductors, whose composition and hybrid form considerably affect their EM properties.
3. Research on EMW absorption mechanisms. Elucidation of EMW absorption mechanisms is the precondition for designing high‐performance MAMs. Progress has been made in terms of clarifying the EMW absorption mechanisms in optimization‐engineering‐based texture adjustment approaches. However, studies on the effective responses elicited by various structural features are still limited. Generally, topological manipulation is related to the optimization of impedance matching and the construction of electronic conductive loops, whereas defects and heterointerfaces are typically related to polarization relaxation. Therefore, in addition to the demand for more microscopic mechanistic explorations, adopting approaches, such as cross‐fusion can be crucial. For instance, in certain magnetic particles, defects can also significantly influence magnetism, as both the anisotropy and size of the magnetic domains can be controlled by the defects. In this regard, fundamental research on EMW absorption is anticipated to make major breakthroughs based on the texture regulation of MOFs.
4. Advanced characterization and simulation. Advanced characterization and simulation techniques can assist in performing effective structural and performance analyses of MAMs. Structural characterization techniques and theoretical discussions in this context remain in their infancy, because most research is conducted using relatively traditional characterization techniques, such as XRD, SEM, TEM, Raman spectroscopy, and XPS. Fortunately, several advanced characterization techniques and theoretical simulations are being proposed to construct structure/EM‐response models. As mentioned in Section 3, Rietveld Refinement of XRD patterns can be performed to analyze the topology, defects, and hybrid features via strategies, such as simulation of unit cell size, anisotropy, and deletion or substitution of occupancies. However, these simulations are appropriate only for the analysis of the dielectric properties determined by the charge‐transport activity, with only a few studies being conducted on active magnetic sites. Furthermore, charge density profiles, density of states, magnetic domain evolution are being gradually visualized using advanced instruments and simulation techniques such as DFT, CST, multiphysics simulations, and finite element analysis. Moreover, electrochemical activity can also be exploited to establish mechanistic correlations with EMW absorption properties.
5. Practical application. At the moment, most of the current development of MOFs‐derived MAMs is laboratory‐scale. However, it is supposed to attain an industrial scale and achieve practical application. The most critical point is that we need to increase the yield of MOFs precursors and reduce their industrial costs at first. Most of the current MOFs are in the form of powders, so it is inevitable to prepare MOFs‐derived MAMs with high mechanical properties, such as films, fabrics, fibers, gels, and foams. In order to adapt to diverse and harsh practical application scenarios, such as high altitude or deep sea, these materials should also hold the characteristics of high durability, corrosion resistance, low density, sustainable, abrasion resistance, high and low temperature resistance, and transparency. Ultimately, in this era of rapid development, it will be a promising field to develop multifunctional and intelligent devices based on MOFs derived MAMs.

The past decade has witnessed encouraging progress in novel texture regulation of MOFs for structural optimization engineering of MAMs. Although promising, there is clearly a lot of work waiting to be done. This review provides new enlightening perspectives for developing highly efficient MAMs and accelerating their actual implementation not only via synthesis strategies but also in terms of elucidating the EMW absorption mechanisms. This review is anticipated to spur innovation in the development of MOF‐derived MAMs to overcome the bottleneck of structural design of MOFs in the field of EMW‐absorbing materials.

## Conflict of Interest

The authors declare no conflict of interest.
